# Enhanced chondrogenesis of bone marrow-derived stem cells by using a combinatory cell therapy strategy with BMP-2/TGF-β1, hypoxia, and *COL1A1*/*HtrA1* siRNAs

**DOI:** 10.1038/s41598-017-03579-y

**Published:** 2017-06-13

**Authors:** Florence Legendre, David Ollitrault, Tangni Gomez-Leduc, Mouloud Bouyoucef, Magalie Hervieu, Nicolas Gruchy, Frédéric Mallein-Gerin, Sylvain Leclercq, Magali Demoor, Philippe Galéra

**Affiliations:** 1 Caen Normandy University, France; UNICAEN EA7450 BioTARGen (Biologie, Génétique et Thérapies ostéoArticulaires et Respiratoires), 3 rue Nelson Mandela, 14280 Saint-Contest, France; 2 Laboratoire de Cytogénétique Prénatale, Service de Génétique, CHU Caen, France; 3 Institute for Biology and Chemistry of Proteins, CNRS, UMR 5305 Laboratory of Tissue Biology and Therapeutic Engineering, Université Claude Bernard-Lyon 1 and University of Lyon, Lyon, France; 4 Service de Chirurgie Orthopédique, Clinique Saint-Martin, Caen, France

## Abstract

Mesenchymal stem cells (MSCs) hold promise for cartilage engineering. Here, we aimed to determine the best culture conditions to induce chondrogenesis of MSCs isolated from bone marrow (BM) of aged osteoarthritis (OA) patients. We showed that these BM-MSCs proliferate slowly, are not uniformly positive for stem cell markers, and maintain their multilineage potential throughout multiple passages. The chondrogenic lineage of BM-MSCs was induced in collagen scaffolds, under normoxia or hypoxia, by BMP-2 and/or TGF-β1. The best chondrogenic induction, with the least hypertrophic induction, was obtained with the combination of BMP-2 and TGF-β1 under hypoxia. Differentiated BM-MSCs were then transfected with siRNAs targeting two markers overexpressed in OA chondrocytes, type I collagen and/or HtrA1 protease. siRNAs significantly decreased mRNA and protein levels of type I collagen and HtrA1, resulting in a more typical chondrocyte phenotype, but with frequent calcification of the subcutaneously implanted constructs in a nude mouse model. Our 3D culture model with BMP-2/TGF-β1 and *COL1A1*/*HtrA1* siRNAs was not effective in producing a cartilage-like matrix *in vivo*. Further optimization is needed to stabilize the chondrocyte phenotype of differentiated BM-MSCs. Nevertheless, this study offers the opportunity to develop a combinatory cellular therapy strategy for cartilage tissue engineering.

## Introduction

Cartilage engineering based on autologous chondrocyte implantation (ACI) offers hope for the treatment of cartilage lesions^1^. ACI, based on Brittberg’s procedure, consists of the harvesting of autologous chondrocytes by biopsy and their expansion *in vitro*, followed by their implantation into the defect under a specialized membrane^2^. Three generations of ACI, using autologous chondrocytes, have contributed to the improvement of this technique. Despite their relative success, other sources of cells have been considered for subsequent generations to overcome complications related to the use of chondrocytes, including poor recovery, the invasiveness of the process, and dedifferentiation during amplification^1^. MSCs are amongst the most promising alternatives to chondrocytes.

MSCs as multipotent stem cells have the ability to self-renew and intrinsically repair and regenerate the tissue in which they reside. They are mostly present in bone marrow, but are also found in various tissues including adipose tissue, the periosteum, muscle, synovial membranes (SM), umbilical cord blood (UCB), and dental pulp. MSCs are characterized by the expression of specific cell surface markers, plastic adherence, and their ability to differentiate into adipocytes, chondrocytes, and osteoblasts under appropriate stimulation *in vitro* ^3, 4^. Their chondrogenic potential varies depending on the source^5^, but other factors, such as the use of biomaterials, growth factors, oxygen pressure, and mechanical strain, must be optimized to control the chondrocyte differentiation process^1^. Most studies on the chondrogenic potential of MSCs^4, 6^ have focused on MSCs from bone marrow (BM-MSCs), both for historical reasons and because of their easy access. TGF-β3 appears to be the most promising growth factor for chondrogenesis^7^, although TGF-β1, -β2, and -β3 can all induce chondrogenesis of MSCs. Therefore, TGF-β1 alone is also a potent inducer of MSC chondrogenesis in various scaffolds^8–10^ and in pellet culture system^6^. Members of the bone morphogenetic protein (BMP) family effectively induce chondrogenesis of synovium-derived MSCs in alginate^11^. It is generally agreed that BMPs enhance the chondrogenic effects of TGF-β^12, 13^, but the results on their effect alone are contradictory, in particular for BMP-2^14–16^.

The major challenge of using MSCs as a cell source for cartilage engineering is to prevent the MSC-derived chondrocytes from undergoing hypertrophic maturation, characterized by up-regulation of type X collagen, MMP-13, and ALP activity^17^. Several studies suggest that hypoxia may influence this process and suppress terminal chondrocyte differentiation^1, 18–20^, but others suggest that hypoxia also strongly inhibits *in vitro* chondrogenesis in adipose derived-MSCs and BM-MSCs^21, 22^. In addition, *in vitro* chondrogenesis of MSCs promotes induction of fibrocartilage-like features, such as expression of type I collagen. Gene therapy (e.g. RNA interference) as a strategy to abolish type I collagen expression, may be useful for the chondrogenic differentiation of MSCs^1, 23^. The catabolism of the extracellular matrix (ECM) is elevated in cartilage from osteoarthritis (OA) patients and several proteases show increased expression, including high temperature requirement factor A1 (HtrA1)^24, 25^. This serine protease is highly involved in the proteolysis of aggrecan and the degradation of TGF-β receptor family proteins, required for MSC chondrogenesis^26, 27^. Therefore, the knock-down of HtrA1 may both favor and stabilize TGF-β-enhanced ECM production.

We have already successfully transfected dedifferentiated chondrocytes with small interfering RNAs (siRNAs), leading to prolonged *COL1A1* knock-down in mouse chondrocytes embedded in an agarose hydrogel and in human chondrocytes seeded in a collagen sponge and cultured with BMP-2^28–30^. We have also demonstrated that the combination of BMP-2 (50 ng/mL), hypoxia (3% O_2_), collagen sponge, and siRNAs targeting *COL1A1* and *HtrA1* improved the differentiated phenotype of human chondrocytes both *in vitro* and *in vivo* ^28, 30^. The aim of the present study was to determine the best culture conditions to induce chondrogenesis of human BM-MSCs from aged OA donors and to improve their differentiated phenotype with a knock-down strategy using *COL1A1* and *HtrA1* siRNAs. We analyzed the proliferation potential and plasticity of hBM-MSCs after characterization of their immunophenotype. Next, we cultured hBM-MSCs in type I/III collagen sponge scaffolds under normoxia or hypoxia, with BMP-2 (50 ng/mL) and/or TGF-β1 (10 ng/mL). We used TGF-β1 rather than TGF-β3 because we already induced chondrogenic differentiation of umbilical cord blood (UCB)-derived MSCs with the combination of BMP-2 and TGF-β1 at the same concentrations^31^ and we did not find any real differences in the hBM-MSC chondrogenesis with either TGF-β1 or TGF-β3 (data not shown). In this study, we demonstrated that the best chondrogenic induction, with the least hypertrophy and no osteoblastic maturation, was obtained under hypoxia using the combination of BMP-2 and TGF-β1. We successfully improved the differentiation process to produce cells with a more typical chondrocyte phenotype using siRNAs, but our BMP-2/TGF-β1 3D culture model did not prevent osteoblast maturation of hBM-MSCs implanted subcutaneously into *nude* mice. However, this study provides new insights on the possibility of combining cell therapy and siRNA strategy for cartilage repair in humans.

## Materials and Methods

### hBM-MSC isolation and culture

Bone marrow was obtained from the iliac crests of patients undergoing total hip replacement for osteoarthritis (age range: 53–85 years, median 69 years; 56% male and 44% female). All patients signed an informed consent agreement form. All experiments with human samples from the Orthopedic Surgery Department of St Martin Clinic (Caen, France) were approved by the local Ethics Committee for research with human samples (Comité de Protection des Personnes Nord Ouest III) of the “Centre Hospitalier Universitaire” of Caen. All methods were carried out in accordance with relevant guidelines and regulations. The aspirates were washed once with phosphate buffered saline (PBS; Gibco, Life Technologies, Saint-Aubin, France) and centrifuged at 400 × g for 10 min. The cell pellet was resuspended in growth medium consisting of alpha-modified essential medium (α-MEM; Gibco, Life Technologies) supplemented with 10% fetal calf serum (Gibco, Life Technologies), 2 mM L-glutamine, 1 ng/mL FGF-2 (Sigma-Aldrich, Saint-Quentin Fallavier, France), and antibiotics (100 IU/ml penicillin, 100 μg/mL erythromycin, and 0.25 μg/mL fungizone). BM-MSCs were isolated by plastic adhesion and successive passages in tissue culture flasks incubated at 37 °C in a 5% CO_2_ atmosphere. After 3 days, non-adherent cells were discarded, and adherent BM-MSCs were cultured to confluence, changing the growth medium twice a week. BM-MSCs were harvested by trypsinization (0.25% trypsin/1 mM EDTA, Gibco, Life Technologies), seeded at 1 × 10^4^ cells/cm^2^, and amplified to the desired passage.

### Cumulative population doublings

hBM-MSCs were trypsinized every 7 days from P2 to P7 and the cells counted using a hemocytometer. The population-doubling level was calculated, according to the equation: population doublings (PD) = [log10(NH) − log10(NS)]/log10(2), where NH is the number of harvested cells and NS the number of seeded cells. Then, the cumulative population-doubling level (CPD) was obtained by adding the PD levels of the previous passages: CPD = ΣPD.

### Immunophenotyping

BM-MSCs (from 8 donors between P3 and P7) were harvested, washed, and resuspended in PBS-sodium azide buffer (0.1%) at a density of 10 × 10^6^ cells/ml. Cell suspensions (0.5 × 10^6^ cells/50 µl) were incubated with 3 to 5 µl of monoclonal antibodies for 25 min at 4 °C in the dark. The following anti-human antibodies were used: CD14-fluorescein isothiocyanate (FITC; BD Biosciences), CD34-allophycocyanin (APC; BD Biosciences), CD45-peridinin chlorophyll protein complex (PerCP; BD Biosciences), CD64-phycoerythrin (PE; BD Biosciences), CD73-PE (BD Pharmingen), CD146-PE (BD Pharmingen), HLA-DR-FITC (BD Biosciences), CD29-FITC (Beckman Coulter), CD44-PE (Beckman Coulter), CD90-PE (Beckman Coulter), and CD105-FITC (ABd Serotec). The respective mouse isotype antibodies served as controls. The cells were then washed and resuspended in 300 μL PBS-azide. Acquisitions were performed in a BD FACS Canto II flow cytometer (BD Biosciences) and analyzed using FlowJo Software (TreeStar).

### Osteogenic and adipogenic differentiations

Passage 4 (P4) hBM-MSCs were recovered for osteogenic and adipogenic differentiations of BM-MSCs. The cells were plated at 1 × 10^5^ cells/well in 6-well plates and differentiations were then performed at P5 for 21 days, when cells reached 70% confluence. All reagents used in the differentiation experiments where purchased from Sigma-Aldrich.

Osteogenic medium consisted of α-MEM supplemented with 2 mM L-glutamine, 10% FCS, 10^−7^ M dexamethasone, 100 μM ascorbic acid-2-phosphate, 10 mM β-glycerol phosphate, and antibiotics. The medium was changed twice per week. Osteogenic differentiation was assessed by alizarin red S staining. Briefly, cells were fixed with 4% paraformaldehyde for 10 min at room temperature, washed once with PBS (pH 4.1), and stained for 20 min with 2% w/v alizarin red S at room temperature.

Adipogenic differentiation was obtained by performing three cycles of culture in induction and maintenance medium (for 3 days and 1 day, respectively), followed by 7 days of culture in maintenance medium. The induction medium was composed of α-MEM supplemented with 2 mM L-glutamine, 10% FCS, 1 µM dexamethasone, 0.5 mM 3-isobutyl-1-methyl-xanthine, 100 µM indomethacin, and 10 μg/mL recombinant human (rh) insulin, and antibiotics. The maintenance medium consisted of α-MEM supplemented with 10% FCS, 10 μg/µL insulin, and antibiotics. Intracellular accumulation of lipid vacuoles was visualized using oil red O staining and identified by their bright red color. Briefly, cells were fixed as previously, washed, and stained for 20 min with 0.3% w/v oil red O at room temperature.

Cells were cultured in α-MEM supplemented with only 2 mM L-glutamine, 10% FCS, and antibiotics as negative controls for osteogenic and adipogenic differentiation.

### Chondrogenic differentiation

P4 hBM-MSCs were recovered and used for chondrogenic differentiation at P5 in 3D collagen scaffolds manufactured by Symatèse Biomatériaux (Chaponost, France). These collagen sponge scaffolds were composed of native type I collagen (90–95%) and type III collagen (5–10%) from calf skin. Collagen sponges, 2 mm thick and 5 mm in diameter, were cross-linked using glutaraldehyde to increase their stability and sterilized by ß irradiation. hBM-MSCs were seeded onto the sponges (5 × 10^5^ cells/sponge, *i*.*e*. 15 × 10^6^ cells/cm^3^) in 96-well culture plates and incubated at 37 °C and 5% CO_2_ in incomplete chondrogenic medium (ICM). This medium was composed of high glucose-Dulbecco’s modified eagle medium (DMEM; 4.5 g/L) supplemented with antibiotics, 50 μg/mL ascorbic acid-2-phosphate, 1 mM sodium pyruvate, 40 μg/mL proline (Fluka), 10^−7^ M dexamethasone, and a 1:100 dilution of insulin transferrin selenium supplement (Invitrogen). After 1 h, each cell construct was transferred to 24-well plates and incubated in ICM pre-equilibrated or not to 3% O_2_ by bubbling, and supplemented or not with 50 ng/mL rhBMP-2 (inductOs, Wyeth Europa Ltd.), or 10 ng/mLrhTGF-β1 (Miltenyi Biotec), or both. This point was defined as day 0 and CSMs were incubated under normoxia (21% O_2_) or hypoxia (3% O_2_) for 3, 7, 14, and 21 days. Hypoxic cultures, including any handling, were exclusively performed in a sealed chamber, with a controlled rate of oxygenation. The medium was changed on days 0, 3, 7, 10, 14, and 17, and the sponges harvested on days 0, 3, 7, 14 and 21.

The cell pellets of trypsinized P4 hBM-MSCs were used as controls (day zero, D0) for real time RT-PCR and Western blot experiments.

### Gene silencing experiments

P4 hBM-MSCs were recovered and seeded in collagen sponges at P5. They were cultured with ICM ± BMP-2/TGF-β1 under hypoxia as described above. The medium was changed on days 0, 3, 7, and 10. On days 0 and 7, MSCs were transfected with a mix of INTERFERin (Polyplus-transfection SA, 3 µL), OptiMEM (Gibco, Life Technologies, 100 μL) and siRNAs (100 nM). The siRNAs specifically targeted the *COL1A1* mRNA (target sequence: 5′-ACCAATCACCTGCGTACAGAA-3′, Eurogentec), and the *HtrA1* mRNA (target sequence: 5′-CGGCCGAAGTTGCCTCTTTT-3′, Eurogentec). A negative control siRNA was also used (NC; target sequence: 5′-AATTCTCCGAACGTGTCACGT-3′, Qiagen). The sponge constructs were harvested on day 14.

### Real time RT-PCR analysis

Total RNA was extracted using TRIzol Reagent® (Invitrogen) and 1 µg of DNAse I-treated total RNA was reverse-transcribed into cDNA as previously described^30^. PCRs were performed on an Applied Biosystems 7700 Real-Time system using the TaqMan PCR Master Mix (Applied Biosystems) for *COL2B* and Power SYBR Green PCR (Applied Biosystems) for the other genes, as previously described^28^. Ribosomal protein L13a (RPL13a) was used as an endogenous reference gene. The primers and Taqman probe used in this study are presented in Table S1. Relative gene expression was calculated using the 2^−∆∆Ct^ method or the standard curve method depending on the efficiency of the amplification of RPL13a, and expressed as the mean of triplicate samples.

### Western-blotting

Following treatment, cells cultured in collagen sponges were rinsed once with ice-cold PBS, crushed, and lysed in RIPA buffer to prepare cellular extracts^32^. The cellular extracts underwent SDS-polyacrylamide gel electrophoresis and were electro-transferred to a polyvinylidene difluoride transfer membrane (Millipore). The membranes were probed with type I and II collagens (1:3000 and 1:500 respectively; Novotec), type X collagen (1:1000, Sigma Aldrich), HtrA1 (1:2500, Millipore), and GAPDH (1:2000, Santa Cruz Biotechnology, Inc.) antibodies, and then washed and incubated with secondary peroxidase-conjugated antibodies (1:5000, Santa Cruz Biotechnology, Inc.). The signals were revealed using Western Lightning Plus-ECL (PerkinElmer, Inc.) and the membranes exposed to X-ray film. To obtain control extracts for Western-blot experiments, small cartilage slices from macroscopically healthy zones of the femoral heads of patients undergoing hip replacement were crushed in liquid nitrogen and protein extraction carried out using RIPA buffer. Signals were quantified using Image J software.

### *In vivo* experiments

P4 hBM-MSCs (from 3 OA patients) were recovered and cultured in type I collagen sponges at P5 for 14 days under hypoxia, with or without BMP-2/TGF-β1, and NC, *COL1A1*, or *HtrA1* siRNA, or both *COL1A1* and *HtrA1* siRNAs as described earlier. The sponges were grafted in a subcutaneous location in *nude* mice (Athymics nude-foxn1, female, 4 weeks, Harlan France). Animal experiments were approved by the Regional Ethics Committee (Comité d’Ethique Normandie en matière d’Expérimentation animale, Mont-Saint Aignan, France) (N/01-10-11) and performed in accordance with institutional animal guidelines. Surgical procedures were performed under general anesthesia consisting of inhalation of 4% isoflurane. After 28 days, *nude* mice were euthanized under anesthesia (5% isoflurane) by CO_2_ inhalation. The collagen sponges were recovered and fixed with buffered 4% paraformaldehyde for 24 h.

### Histochemistry

Once fixed, the *in vivo* constructs were dehydrated in successive baths of increasing concentrations of ethanol and embedded in paraffin as previously described with an automaton (Laboratory of Pathological Anatomy, CHU)^30^. Then, 4 µm sections were prepared using a microtome and mounted on silanized slides for immunostaining. The sections were deparaffinized by successive baths of toluene (two baths of 5 min), 100% ethanol (two baths of 5 min), 90% ethanol (5 min), 70% ethanol (5 min) and finally distilled water. The deparaffinized sections were stained with hematoxylin-eosin-safran (HES) and alizarin red S (2%, pH 4.1) according to routine protocols. Samples were mounted with Eukitt (DAKO). An Aperio ScanScope scanner (Leica Biosystems) was used to digitalize the histological slides.

### Statistical analysis

For RT-qPCR analysis, data are generally presented as box plots, representative of four experiments, performed in triplicate. Box plots show the minimum value, the 25^th^, 50^th^ (median), and 75^th^ percentiles, and the maximum values. Means are shown as crosses. The Kruskall-Wallis test was used for multiple comparisons. The Mann-Whitney U test was used to determine significant differences between two groups of treatments (without adjustment for multiple comparisons). The expression of stem cell markers was analysed using Student’s paired *t*-test to determine differences between control cells and MSCs. P values of less than 0.05 were considered to be significant: ***P < 0.001, **P < 0.01 and *P < 0.05.

## Results

### Characterization of hBM-MSCs

**Figure 1:**
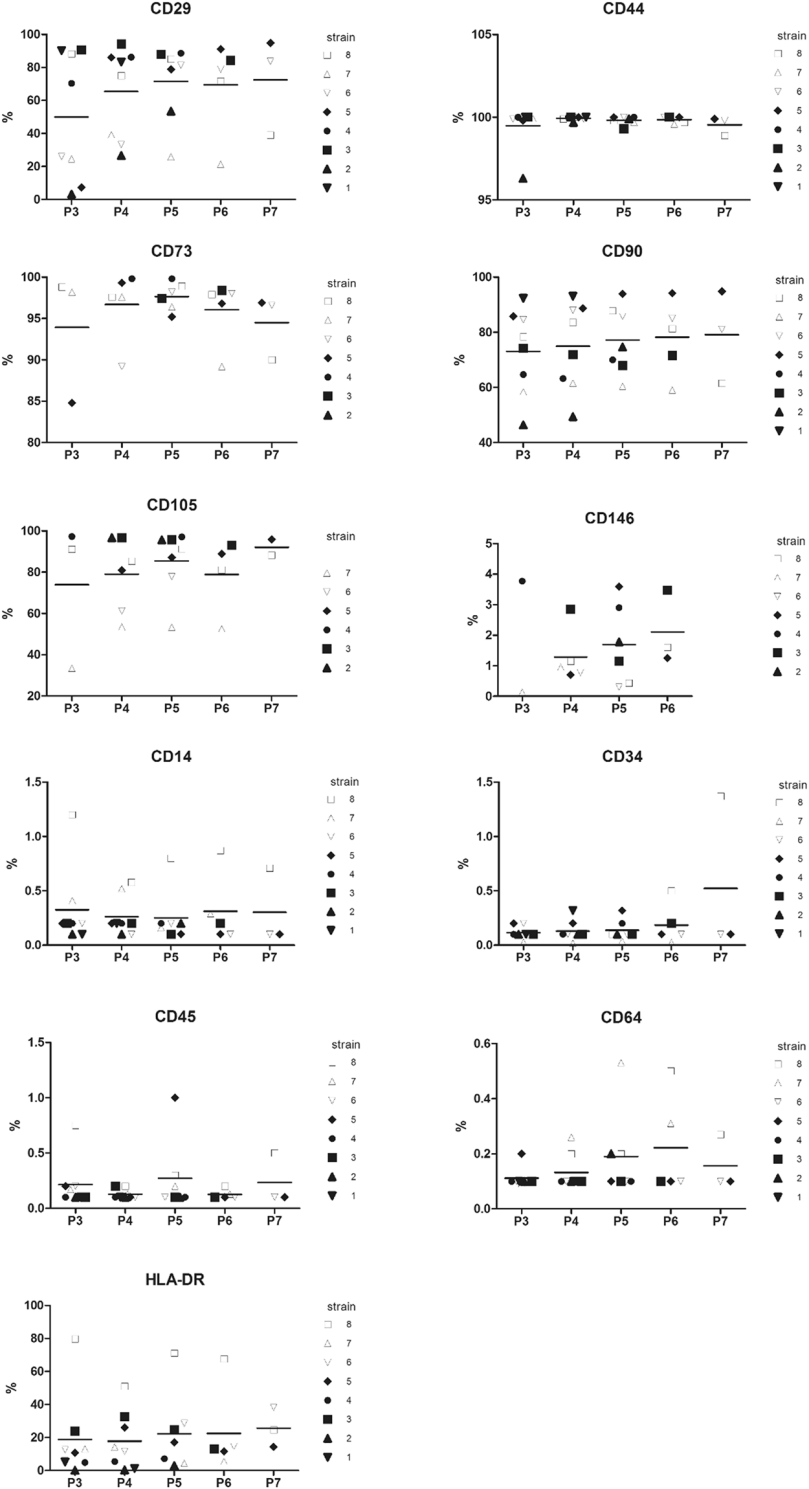
Immunophenotype of hBM-MSCs. The expression of CD29, CD44, CD73, CD90, CD105, CD146, CD14, CD34, CD45, CD64, and HLA-DR was analyzed by flow cytometry in hBM-MSC preparations from eight donors between passage 3 and 7 (n = 8). The graphs show the percentage of positive cells relative to an isotype control depending on the strain and passage. The means are represented by a bar for each passage.

hBM-MSCs from eight OA donors were immunophenotypically analyzed from P3 to P7 (Fig. 1). The results show that hBM-MSCs were all negative for hematopoietic markers (expression < 1% for CD14, CD34, CD45, and CD64), whereas they were positive for several stem cells markers including CD29, CD44, CD73, CD90, and CD105, but with particular features. The expression of CD44 (hyaluronan receptor) was high (>99%) and homogeneous between strains and passages, whereas that of other stem-cell markers was lower and more heterogeneous. The expression of CD29 (integrin-β1) and CD105 (endoglin) appeared to increase during passaging (from 50% positive at P3 to 72% positive at P7 for CD29, and from 74% positive at P3 to 92% positive at P7 for CD105). The expression of CD73 was relatively stable in the population and was higher than 94% during the amplification. CD90 expression (also known as Thy-1), appeared to be donor-dependent, with relatively constant expression between passages for each donor. Approximately 75% of the total population expressed CD90. All hBM-MSCs expressed low levels of CD146 (<4%), a member of the immunoglobulin superfamily, also known as MCAM, MUC18, or Mel-CAM, considered to be a stem cell marker. However, the expression of HLA-DR (human leukocyte antigen), an MHC (major histocompatibility complex) class II molecule, varied depending on the donor (approximately 20% positive in the total population), and cells derived from only 1/3 of the donors did not significantly express HLA-DR (<5%).

**Figure 2:**
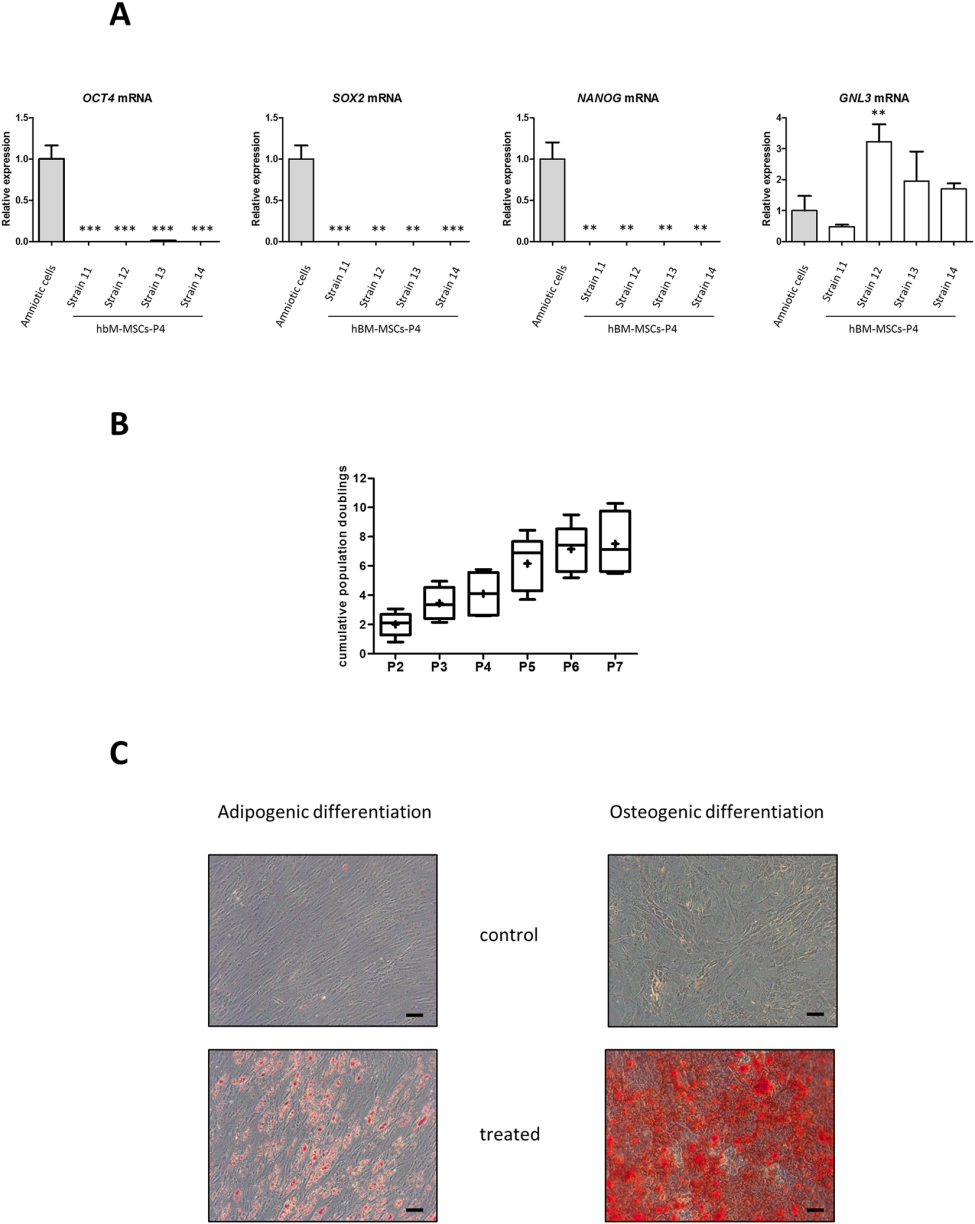
Characterization of hBM-MSCs. **A**. Analysis of pluripotent stem cell markers in hBM-MSCs. The levels of mRNA encoding Oct4 (*OCT4* mRNA), Sox2 (*SOX2* mRNA), Nanog (*NANOG* mRNA), and nucleostemin (*GNL3* mRNA) were measured using real time RT-PCR with specific primers. The results of one experiment performed in triplicate were normalized to *RPL13a* mRNA levels and are presented as the expression of each gene relative to that of amniotic cells (n = 1). Statistically significant differences between amniotic cells and each MSC strain were determined using the Student’s *t*-test ***P < 0.001 and **P < 0.01. **B**. The proliferation of hBM-MSCs was estimated by determining the cumulative population doublings levels (CPD). The CPD of adherent hBM-MSCs were determined between passages 2 and 7 for five donors (strains 5-9) and are represented as box plots (n = 5). **C**. Osteogenic and adipogenic differentiation of P5 hBM-MSCs. Left panels show oil red O staining of hBM-MSCs grown in adipogenic conditions (treated) or not (control) (scale bar: 100 µm). Right panels show alizarin red staining of P5 hBM-MSCs grown in osteoinductive conditions (treated) or not (control) (scale bar: 100 µm). Images shown are representative of three experiments with different donors.

We next analyzed the mRNA expression levels of three key transcription factors essential for the pluripotent and self-renewing phenotypes of embryonic stem cells (ESCs) in the hBM-MSCs (Fig. 2A). The four strains of hBM-MSCs analyzed did not express *OCT4*, *SOX2*, or *NANOG* mRNA (all relative expression < 0.01), unlike amniotic cells. In contrast, they expressed nucleostemin (*GNL3*) mRNA, a stem cell marker which regulates self-renewal and cell cycle progression, at levels similar to those found in amniotic cells.

We examined the proliferation rate of the hBM-MSCs by calculating the CPD with respect to passage number for the cells from five donors (Fig. 2B). We observed an increase in CPD from P2 (2.01 ± 0.8) to P7 (7.5 ± 2.1), but with a low doubling rate between each passage (<1.7 from P2), demonstrating a low rate of proliferation. Moreover, the hBM-MSC proliferation rate decreased with increasing passage number until the cells stopped proliferating between P6 and P7 (doubling rate of 1.05).

We investigated the differentiation potential of hBM-MSCs at P5, first focusing on adipogenic and osteogenic differentiation (Fig. 2C), before focusing on chondrogenic differentiation for the remainder of the study. The adipogenic differentiation of hBM-MSCs was demonstrated by the staining of lipid vacuoles with oil red O only under adipogenic culture conditions. Osteogenic differentiation of hBM-MSCs was also obtained under osteogenic culture conditions as shown by alizarin red staining.

These results show that hBM-MSCs from aged OA donors express adult stem cell markers at different levels (except CD146) and have a reduced proliferative capacity, but can undergo osteogenic and adipogenic differentiation.

### Chondrogenic differentiation and phenotypic profiles of hBM-MSCs

We promoted chondrogenic differentiation of the hBM-MSCs by culturing them in collagen sponge scaffolds in a chondrogenic medium, with BMP-2, TGF-β1 or both, under normoxia or hypoxia for 3, 7, 14 and 21 days, to determine the best conditions of chondrogenesis. We first verified that MSCs colonized the scaffold and had a rounded shape under normoxia or hypoxia (Supplementary Fig. S1).

**Figure 3:**
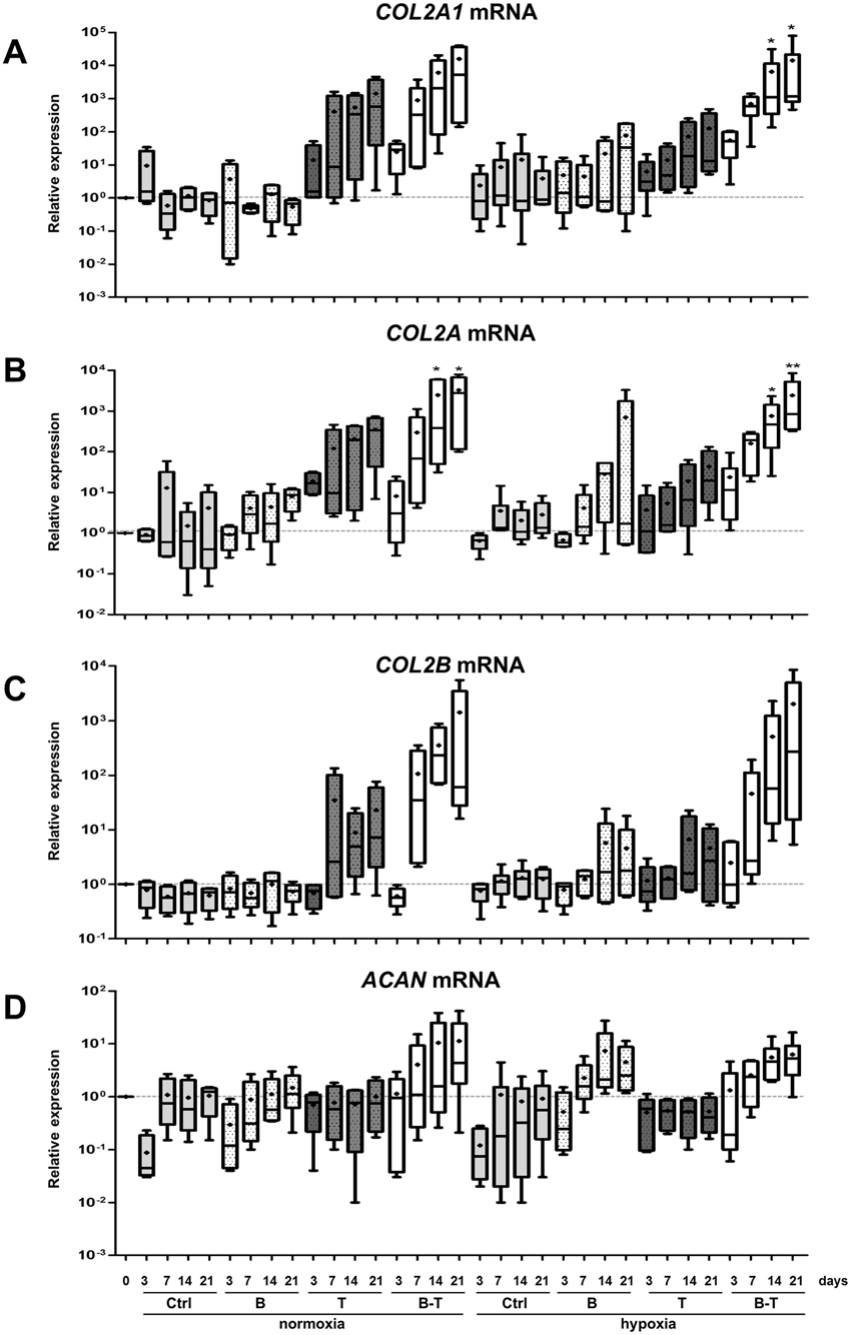
Effects of oxygen tension, BMP-2 and/or TGF-β1 on the mRNA levels of specific cartilage markers. hBM-MSCs were cultured in type I collagen sponges for 3, 7, 14, and 21 days under normoxia or hypoxia, in the absence (Ctrl), or presence of 50 ng/mL BMP-2 (B), 10 ng/mL TGF-β1 (T), or both (B-T). The steady-state mRNA levels for type II collagen were measured using real time RT-PCR with primers or probe specific for type IIA collagen (*COL2A* mRNA) (**B**), type IIB collagen (*COL2B* mRNA) (**C**), or the total amount of type IIA and IIB collagen (*COL2A1* mRNA) (**A**). The levels of mRNA encoding aggrecan (*ACAN* mRNA) were also evaluated with specific primers (**D**). All results were normalized to *RPL13a* mRNA and are presented as the expression of each gene relative to that of undifferentiated hBM-MSCs at day 0. Box plots represent independent experiments performed in triplicate (n = 4). Statistically significant differences between the undifferentiated cells at day 0 and treated cells are presented and were determined using the Kruskall-Wallis test (*P < 0.05, **P < 0.01).

We next determined the differentiation status of hBM-MSCs by measuring the mRNA levels. We first analyzed the steady-state mRNA levels of the two well-known chondrocyte phenotype markers, aggrecan and type II collagen, as well as those of prechondrogenic type IIA collagen and mature type IIB collagen (Fig. 3). The expressions of aggrecan, type II collagen and its spliced variants were not significantly induced by collagen sponge culture (average rates <10 for collagens). The addition of BMP-2 did not improve the expression of these four markers under normoxia, and tended to increase their expression after 14 and 21 days under hypoxia (22- and 77-fold for *COL2A1* mRNA, 2.3- and 5.4-fold for *ACAN* mRNA). However, the addition of TGF-β1 increased *COL2A1*, *COL2A*, and *COL2B* mRNA levels from seven days in culture, and levels seemed to be higher under normoxia than hypoxia (e.g. approximately 0.4 × 10^3^- to 1.4 × 10^3^-fold under normoxia *versus* 14- to 1.3 × 10^2^-fold under hypoxia for *COL2A1* mRNA). TGF-β1 did not modulate *ACAN* expression, regardless of the oxygen levels and incubation times. The combination of both BMP-2 and TGF-β1 had an additive effect on the mRNA levels of all forms of type II collagen, under normoxia or hypoxia from seven days in culture and after. Thus, the BMP-2/TGF-β1 combination greatly increased the mRNA levels of all forms of type II collagen from 7 to 21 days (from approximately 8 × 10^2^- to 1.5 × 10^4^-fold for *COL2A1* mRNA, and from approximately 1 × 10^2^- to 2 × 10^4^-fold for *COL2A* and *COL2B* mRNA). The chondrogenic effect of the BMP-2/TGF-β1 combination was more significant in hypoxia for *COL2A1* mRNA levels at day 14 and 21. The BMP-2/TGF-β1 combination had a smaller effect on *ACAN* mRNA levels with a maximum increase of 11-fold at day 21 under normoxia.

**Figure 4:**
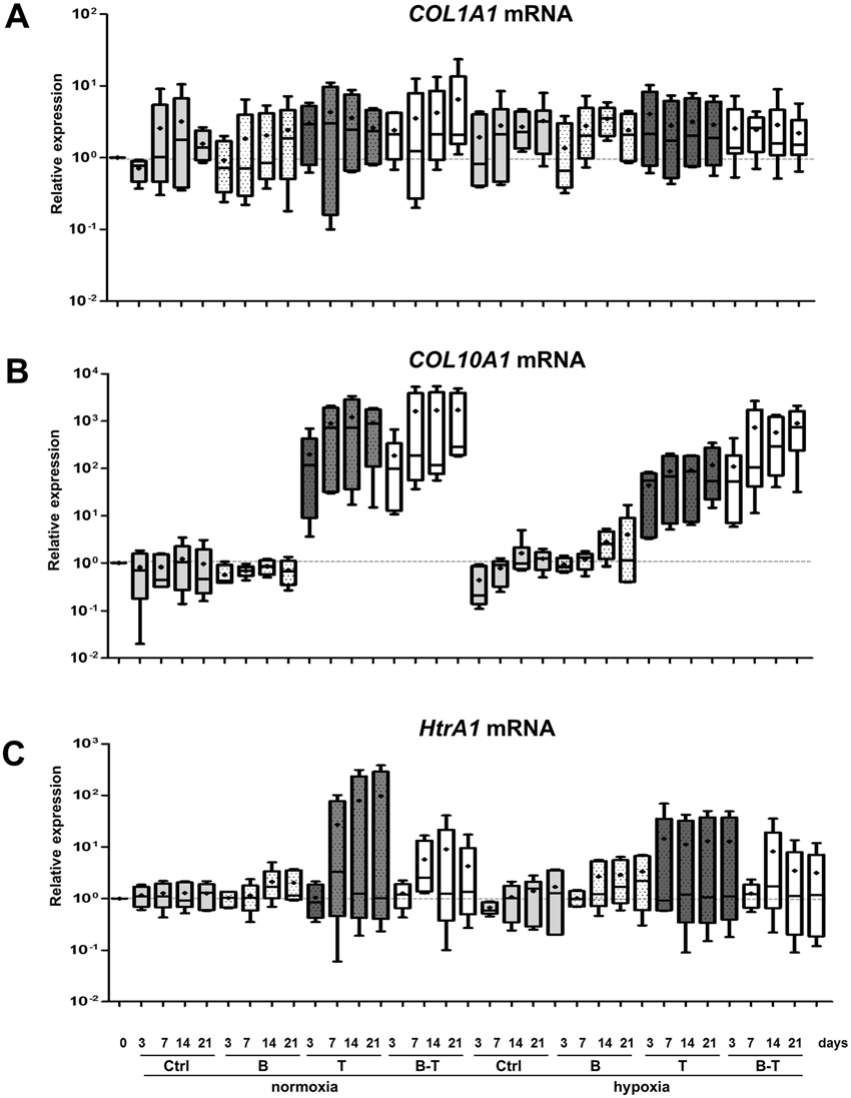
Effects of oxygen tension, BMP-2 and/or TGF-β1 on the mRNA levels of nonspecific cartilage markers and the protease HtrA1. hBM-MSCs were cultured in type I collagen sponges for 3, 7, 14, and 21 days as described in Fig. 3. The levels of mRNA encoding type I collagen (*COL1A1* mRNA) (**A**), type X collagen (*COL10A1* mRNA) (**B**) and HtrA1 (*HtrA1* mRNA) (**C**) were measured using real time RT-PCR with specific primers. All the results were normalized to *RPL13a* mRNA and are presented as the expression of each gene relative to that of undifferentiated hBM-MSCs at day 0. Box plots represent independent experiments performed in triplicate (n = 4). Statistically significant differences between the undifferentiated cells at day 0 and treated cells are presented and were determined using the Kruskall-Wallis test (*P < 0.05).

We also analyzed the expression of type I collagen, the marker of chondrocyte dedifferentiation (Fig. 4A). We found that *COL1A1* mRNA levels remained stable under all culture conditions, and were approximately two times higher than at day 0. As a result, the differentiation index, corresponding to the ratio of type II collagen to type I collagen mRNA (*COL2A1*/*COL1A1* ratio) was higher under hypoxia than normoxia, with the best results at 21 days (Supplementary Fig. S2). The *ACAN*/*COL1A1* ratio was also higher under hypoxia using the BMP-2/TGF-β1combination (Supplementary Fig. S2).

Type X collagen is a marker of chondrocyte hypertrophy. The addition of BMP-2 to 3D scaffold cultures did not significantly influence the expression of *COL10A1* under normoxia or hypoxia (Fig. 4B). However, TGF-β1, or the combination of both BMP-2 and TGF-β1, strongly induced its expression from three days in culture. The expression of *COL10A1* was then relatively stable over seven days. Normoxia seemed to favor TGF-β1-induced transcription of *COL10A1* (approximately 2 × 10^2^- to 1 × 10^3^-fold under normoxia *versus* 5 × 10^1^- to 1 × 10^2^-fold under hypoxia in the presence of TGF-β1). The effect of the BMP-2/TGF-β1 combination on *COL10A1* mRNA levels was also greater under normoxia than hypoxia. The induction of *COL2A1* expression was still higher than that of *COL10A1*, especially under hypoxia with BMP-2/TGF-β1, as shown by the *COL2A1*/*COL10A1* ratio (Supplementary Fig. S2). Moreover, differentiated MSCs did not exhibit osteoblastic maturation in the presence of BMP-2/TGF-β1 at either oxygen tension, as demonstrated by relatively stable *OSTEOCALCIN* mRNA levels (Supplementary Fig. S3).

Finally, we evaluated the steady-state mRNA levels of HtrA1, a protease induced in OA cartilage (Fig. 4C). TGF-β1 exposure tended to increase *HtrA1* expression, particularly under normoxia (approximately 30- to 1 × 10^2^-fold under normoxia from day 7 to 21 *versus* approximately 13-fold under hypoxia from day 3 to 21; not significant because of high standard deviations). The addition of BMP-2 decreased the effect of TGF-β1under normoxia and hypoxia. The expression of *MMP-13*, another protease also considered to be an indicator of hypertrophy, reacted in the same way as that of *HtrA1* (Supplementary Fig. S3). The expression of the protease MMP-1 remained stable in the presence of TGF-β1 or both BMP-2 and TGF-β1 (Supplementary Fig. S3).

In summary, the best induction of chondrocyte-specific phenotypic markers was obtained by culturing the hBM-MSCs in collagen sponges with both BMP-2 and TGF-β1, resulting in a greater and significant effect under hypoxia at day 14 and 21. Moreover, co-treatment with BMP-2 and TGF-β1 did not enhance the expression of *COL1A1*, the marker of chondrocyte dedifferentiation. However, the BMP-2/TGF-β1 combination also induced the expression of the hypertrophic marker *COL10A1*, which was more favored under normoxia (stable from 7 days of culture). The BMP-2/TGF-β1 combination also slightly induced the mRNA expression of the protease HtrA1.

### Synthesis of a specialized ECM by differentiated hBM-MSCs

**Figure 5:**
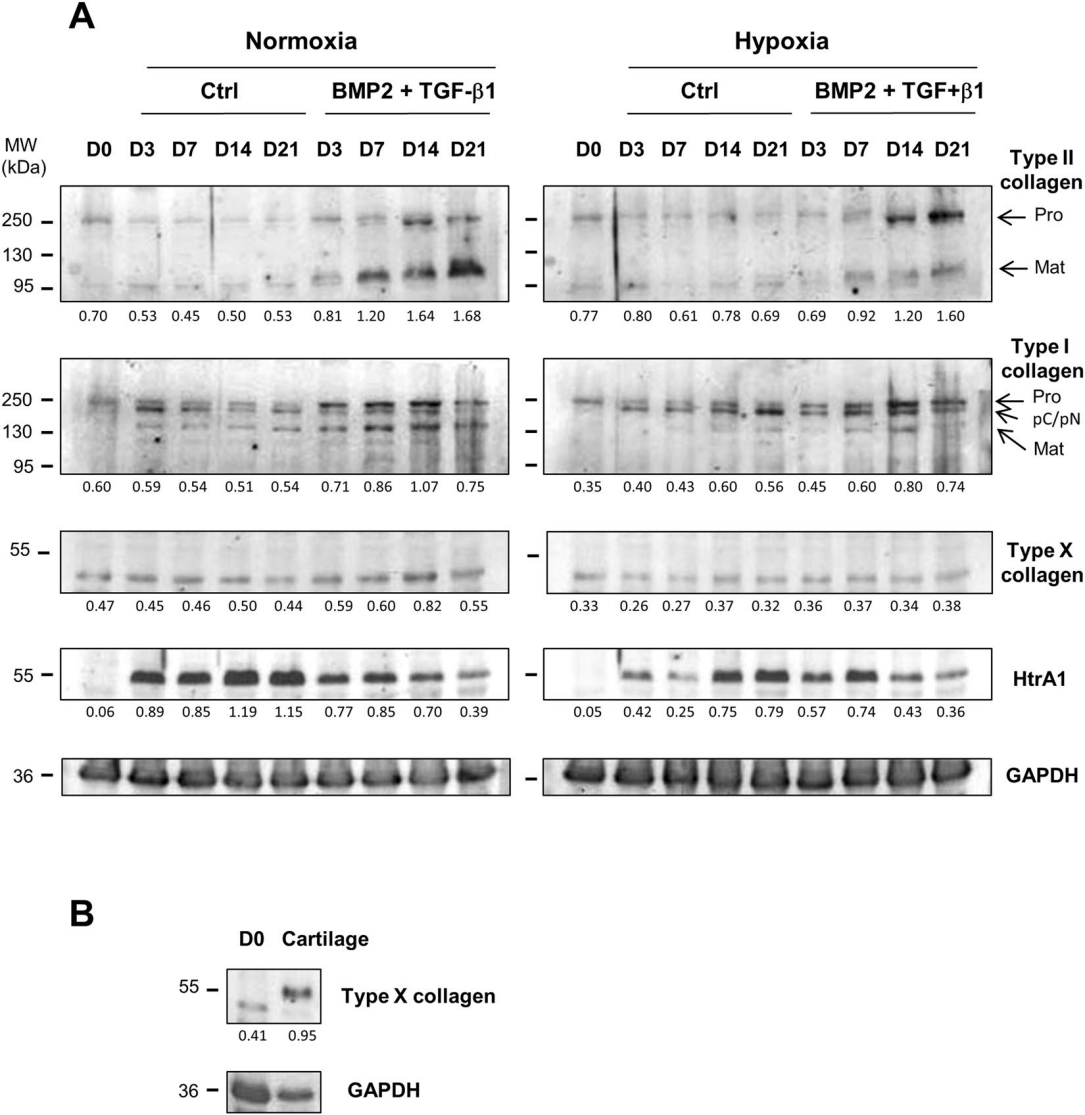
Effect of culture conditions on collagen and HtrA1 synthesis. hBM-MSCs were cultured in type I collagen sponges for 0, 3, 7, 14, and 21 days under normoxia or hypoxia, in the absence (Ctrl), or presence of both 50 ng/mL BMP-2 and 10 ng/mL TGF-β1. Protein extracts were analyzed by Western-blotting for type I, II, and X collagen, and HtrA1 *versus* GAPDH (**A**). Protein extracts from undifferentiated MSCs at day 0 (D0) and a macroscopically healthy zone of human OA cartilage were also analyzed by Western-blotting for type X collagens *versus* GAPDH (**B**). Different exposures of the blots are provided in the supplementary information file (Fig. S5). Corresponding quantifications of the bands of interest, normalized to the GAPDH signals, are shown as arbitrary units below each blot. Representative blots are shown.

We then monitored the chondrogenic effects of BMP-2 and TGF-β1 co-treatment at the protein level. Western-blot analysis showed that only treatment with BMP-2 and TGF-β1 induced the expression of the two isoforms of type II collagen with the maximum effect after 21 days of culture (Fig. 5A). Normoxia favored the appearance of the mature form of type II collagen.

Under normoxia, type I collagen levels gradually decreased during culture, but mature type I collagen forms did not disappear (Fig. 5A). BMP-2/TGF-β1 treatment increased the level of all isoforms of type I collagen until 14 days of culture. Under hypoxia, immature forms of type I collagen predominated and collagen sponge culture increased their expression. The combination of BMP2/TGF-β1 accentuated this increase and also induced the mature form of type I collagen until day 14, but to a lesser extent than under normoxia.

Whatever the culture conditions, hBM-MSCs did not express the 59 kDa form of type X collagen expected in denatured-reduced conditions, whereas this form was expressed in OA cartilage, used as a positive control (Fig. 5A and B). This low molecular-weight form was weakly expressed, and mainly induced at day 14 under normoxia with BMP-2/TGF-β1 (1.7-fold).

The sponge culture strongly induced the expression of the protease HtrA1 (Fig. 5A). Hypoxia delayed this induction relative to normoxia (from 1.5- to 3-fold). BMP-2/TGF-β1 treatment also diminished this induction over the 21 days of culture under normoxia, and only from 14 days of culture under hypoxia.

These results confirm our mRNA data and demonstrate that BMP-2/TGF-β1 treatment, in our 3D culture model, induced chondrogenesis of hBM-MSCs, although type I collagen expression persisted.

### Improvement of the differentiated phenotype with *COL1A1* and/or *HtrA1* siRNAs

We used a siRNA that specifically targets *COL1A1* mRNA to overcome type I collagen expression in hBM-MSCs in the following experiments. We also used a siRNA that targets *HtrA1* mRNA to enhance the action of BMP-2/TGF-β1 and reduce cartilage degradation. Moreover, we performed these experiments under hypoxia to limit the induction of hypertrophy, the expression of HtrA1 and the mature form of type I collagen. HBM-MSCs, seeded onto the sponges, were transfected at days 0 and 7 with a NC, *COL1A1*, or *HtrA1* siRNA, or with both *COL1A1* and *HtrA1* siRNAs, and cultured for 14 days under hypoxia, with or without BMP-2/TGF-β1 treatment, to induce chondrogenesis. We analyzed their phenotypic profile at the transcriptional and protein levels.

**Figure 6:**
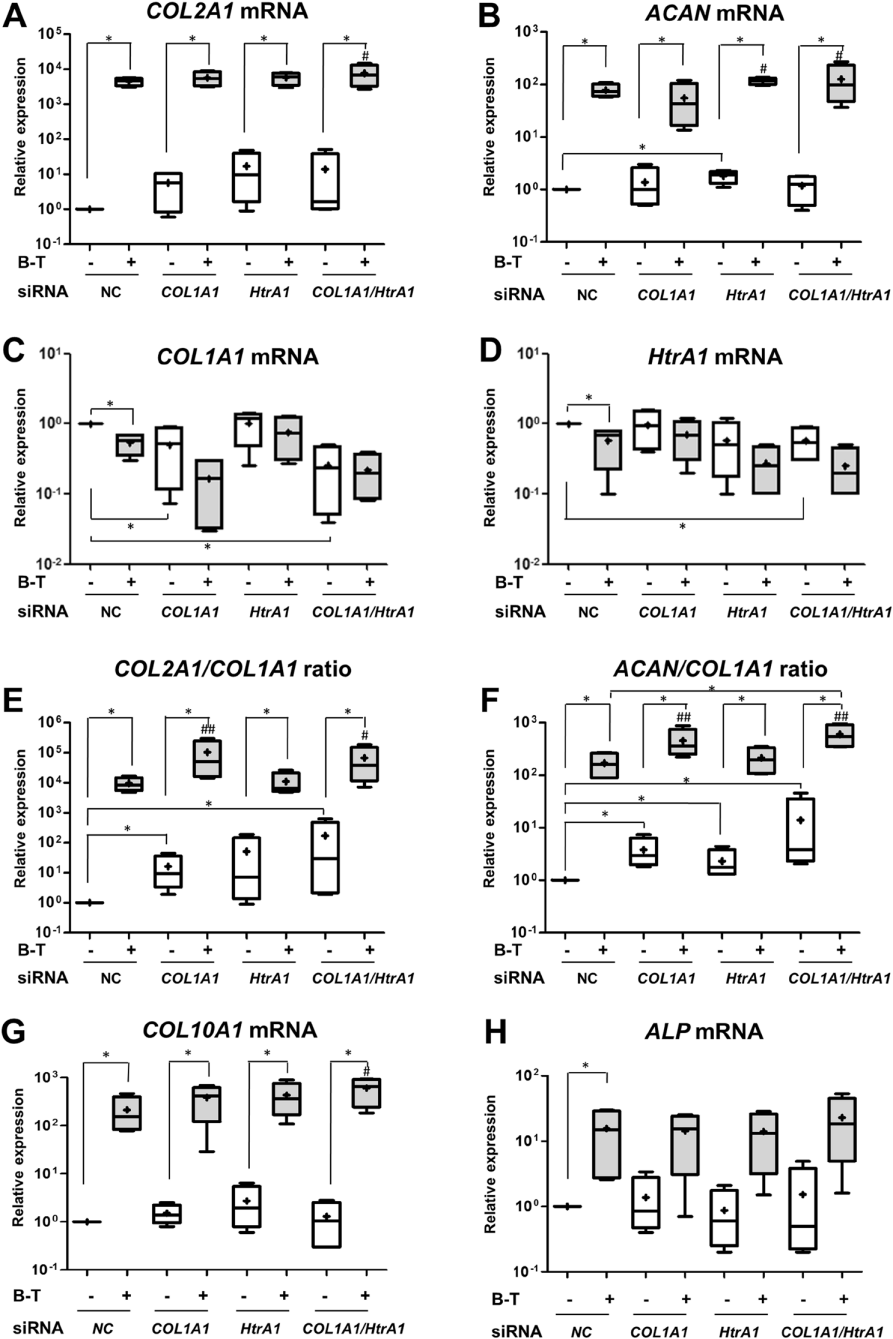
Effects of *COL1A1* and/or *HtrA1* siRNAs on the mRNA levels of specific and nonspecific cartilage markers. hBM-MSCs seeded onto collagen sponges were transfected with an INTERFERin^®^-siRNA complex (100 nM negative control (NC) siRNA, or 100 nM *COL1A1* siRNA and/or 100 nM *HtrA1* siRNA) at days 0 and 7 as described in Materials and Methods. hBM-MSCs were cultured in 3% O_2_ for 14 days with or without (−) both 50 ng/mL BMP-2 and 10 ng/mL TGF-β1 (B-T). Relative mRNA expressions of type II collagen (**A**), aggrecan (**B**), type I collagen (**C**), HtrA1 (**D**), type X collagen (**G**), and alkaline phosphatase (**H**) were obtained as described in Figs 3–4. All results were normalized to *RPL13a* mRNA, and are presented as the expression of each gene relative to that of untreated NC cells. We also determined the *COL2A1*/*COL1A1* mRNA ratio (**E**) and the *ACAN*/*COL1A1* mRNA ratio (**F**). Box plots represent the results from independent experiments performed in triplicate (n = 4). Statistically significant differences between the untreated NC cells and the different treatments were determined using the Kruskall-Wallis test (^#^P < 0.05). Statistically significant differences between untreated cells and BMP-2/TGF-β1-treated cells, NC siRNA-untreated cells, and other siRNA-untreated cells, and between NC siRNA BMP-2/TGF-β1-treated cells and other siRNA BMP-2/TGF-β1-treated cells were determined using the Mann-Whitney U test (*p < 0.05).

BMP-2/TGF-β1 treatment strongly increased *COL2A1* mRNA levels (4.5 × 10^3^ with the NC siRNA treatment; Fig. 6A) and, to a lesser extent, *ACAN* mRNA levels (79-fold, Fig. 6B), as expected. In parallel, BMP-2/TGF-β1 treatment induced a two-fold decrease of *COL1A1* and *HtrA1* mRNA levels in the presence of the NC siRNA (Fig. 6C and D). *COL1A1* and *HtrA1* siRNAs inhibited their specific target with or without BMP-2/TGF-β1 treatment. We observed a 50% decrease of *COL1A1* mRNA levels by the *COL1A1* siRNA in the absence of BMP-2/TGF-β1 (relative to untreated cells with NC siRNA). This decrease was accentuated by BMP-2/TGF-β1 treatment. We also observed a 43% decrease of *HtrA1* mRNA levels by *HtrA1* siRNA in untreated cells. However, there were no statistically significant differences between NC siRNA and *COL1A1* or *HtrA1* siRNA in BMP-2/TGF-β1-treated cells because of high standard deviations and the already negative effects of BMP-2/TGF-β1 treatment by itself. The combination of both siRNAs, with or without BMP-2/TGF-β1, had no more effect than each siRNA alone on their own target. We also did not observe significant differences for the mRNA levels of aggrecan (Fig. 6B) and all forms of type II collagen (Supplementary Fig. S4) between NC siRNA BMP-2/TGF-β1-treated cells and the other siRNAs BMP-2/TGF-β1-treated cells. Nevertheless, the association of BMP-2/TGF-β1 treatment and *COL1A1*/*HtrA1* siRNAs induced the greatest increase of *COL2A1* and *ACAN* mRNA levels (Fig. 6A and B). Moreover, the association of BMP-2/TGF-β1 treatment and the *COL1A1* siRNA or the combination of both siRNAs increased the *COL2A1*/*COL1A1* and *ACAN*/*COL1A1* ratio (Fig. 6E and F). We also verified that the various siRNA treatments did not influence the expression of the proteases MMP-1 and MMP-13, in the presence or absence of BMP-2/TGF-β1 treatment (Supplementary Fig. S4).

As described earlier, BMP-2/TGF-β1 highly induced *COL10A1* mRNA expression (2.1 × 10^2^-fold for the NC siRNA treatment, Fig. 6G). *COL1A1* siRNA, *HtrA1* siRNA, and in particular, the combination of both siRNAs appeared to slightly increase this induction (from 1.8- to 2.8-fold relative to NC siRNA BMP-2/TGF-β1 treated cells). In the presence of NC siRNA, BMP-2/TGF-β1 induced a 15-fold increase in *ALP* mRNA levels. We observed the same effect for all siRNA treatments. However, the mRNA levels of *OSTEOCALCIN* were not modified by any treatment (Supplementary Fig. S4).

**Figure 7:**
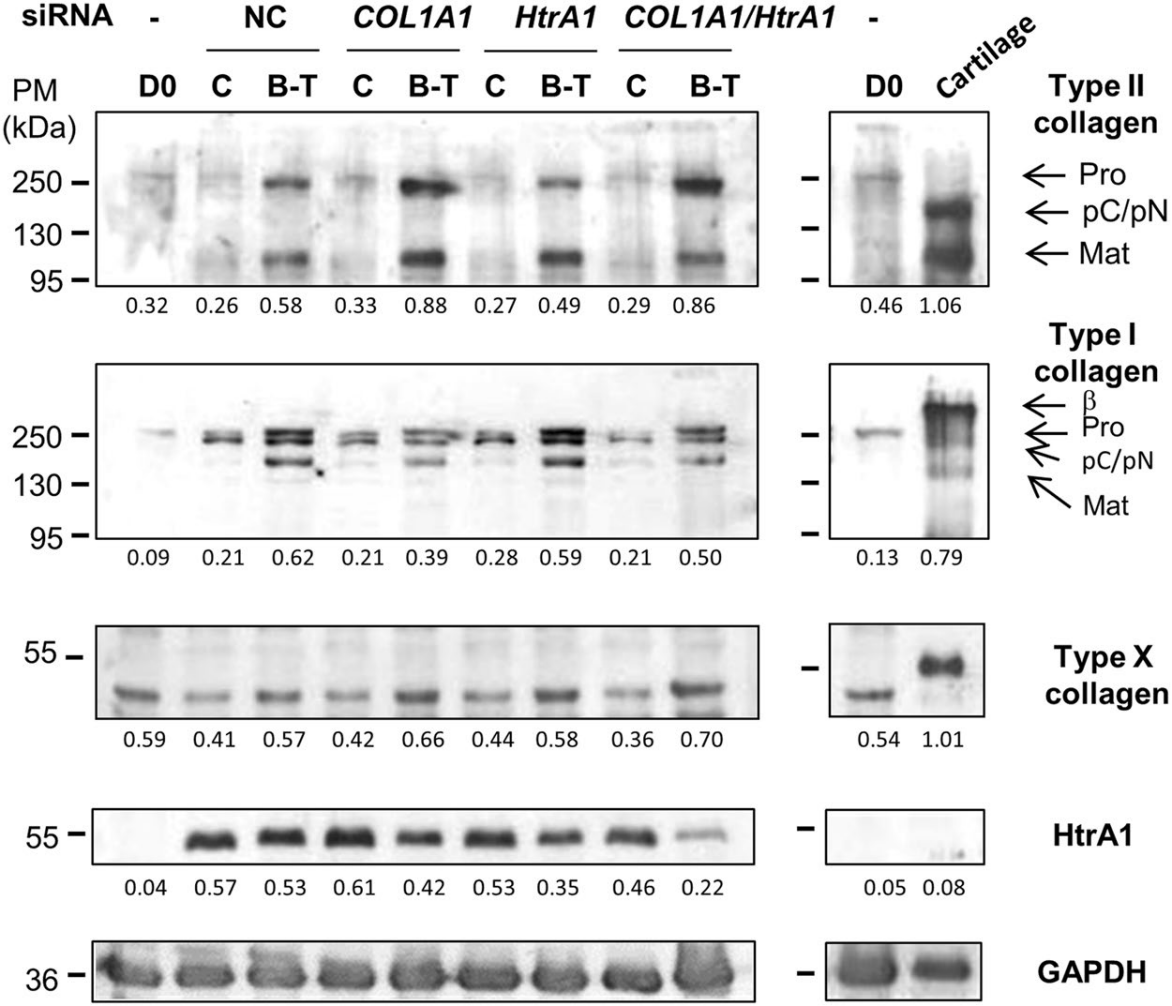
Effects of *COL1A1* and/or *HtrA1* siRNA on collagen and HtrA1 synthesis. hBM-MSCs were cultured under hypoxia with or without BMP-2/TGF-β1 (B-T), and transfected with NC, or *COL1A1* and/or *HtrA1* siRNAs in type I collagen sponges for 14 days, as described in Fig. 6. Protein extracts from treated-MSCs, undifferentiated MSCs at day 0 (D0), and a macroscopically healthy zone of human OA cartilage were analyzed by Western-blotting for type I, II, and X collagens, and HtrA1 *versus* GAPDH. Different exposures of the blots are provided in the supplementary information file (Fig. S6). Corresponding quantifications of the bands of interest, normalized to the GAPDH signals, are indicated as arbitrary units below each blot. Representative blots are shown.

Western-blots performed in parallel showed that BMP-2/TGF-β1 treatment, in the presence of NC siRNA, increased type I and type II collagen synthesis, without significantly affecting HtrA1 production, which was induced by 3D culture (as described earlier) (Fig. 7). *COL1A1* siRNA, in the presence of BMP-2/TGF-β1 treatment, significantly decreased all forms of type I collagen (40% lower than in NC siRNA BMP-2/TGF-β1-treated cells) and, at the same time, amplified (+51%) BMP-2/TGF-β1-induced type II collagen synthesis (procollagen and mature collagen). *COL1A1* siRNA had no further effect on basal HtrA1 synthesis, but we observed a decrease of its synthesis with the addition of BMP-2/TGF-β1 (−26%). *HtrA*1 siRNA significantly down-regulated HtrA1 synthesis in the presence of BMP-2/TGF-β1 (34% lower than in NC siRNA BMP-2/TGF-β1-treated cells), without affecting total type I and type II collagen syntheses. The combination of both siRNAs had no further effect on type I and II collagen production than *COL1A1* siRNA alone. However, co-treatment with both siRNAs decreased HtrA1 synthesis more than *HtrA1* siRNA alone, especially in the presence of BMP-2/TGF-β1 (−59% versus −34%). OA cartilage also showed a high amount of type I, X, and II collagens, but we observed no synthesis of HtrA1. Additionally, *COL1A1* siRNA and the combination of both siRNAs slightly increased the synthesis of low molecular-weight form of type X collagen induced by BMP-2/TGF-β1 treatment (+15% and +22%, respectively, relative to NC siRNA BMP-2/TGF-β1-treated cells).

*COL1A1* siRNA, or the combination of both *COL1A1* and *HtrA1* siRNAs, improved the response of hBM-MSCs to BMP-2/TGF-β1 treatment by decreasing the synthesis of type I collagen and HtrA1 in favor of type II collagen. These results suggest that the association of the two siRNAs could improve chondrogenesis in hBM-MSCs while limiting the catabolic pathway at the same time. However, chondrocytes derived from hBM-MSCs under our experimental conditions expressed a low molecular-weight form of type X collagen.

### Behavior of the neo-cartilage formed *in vivo*

**Figure 8:**
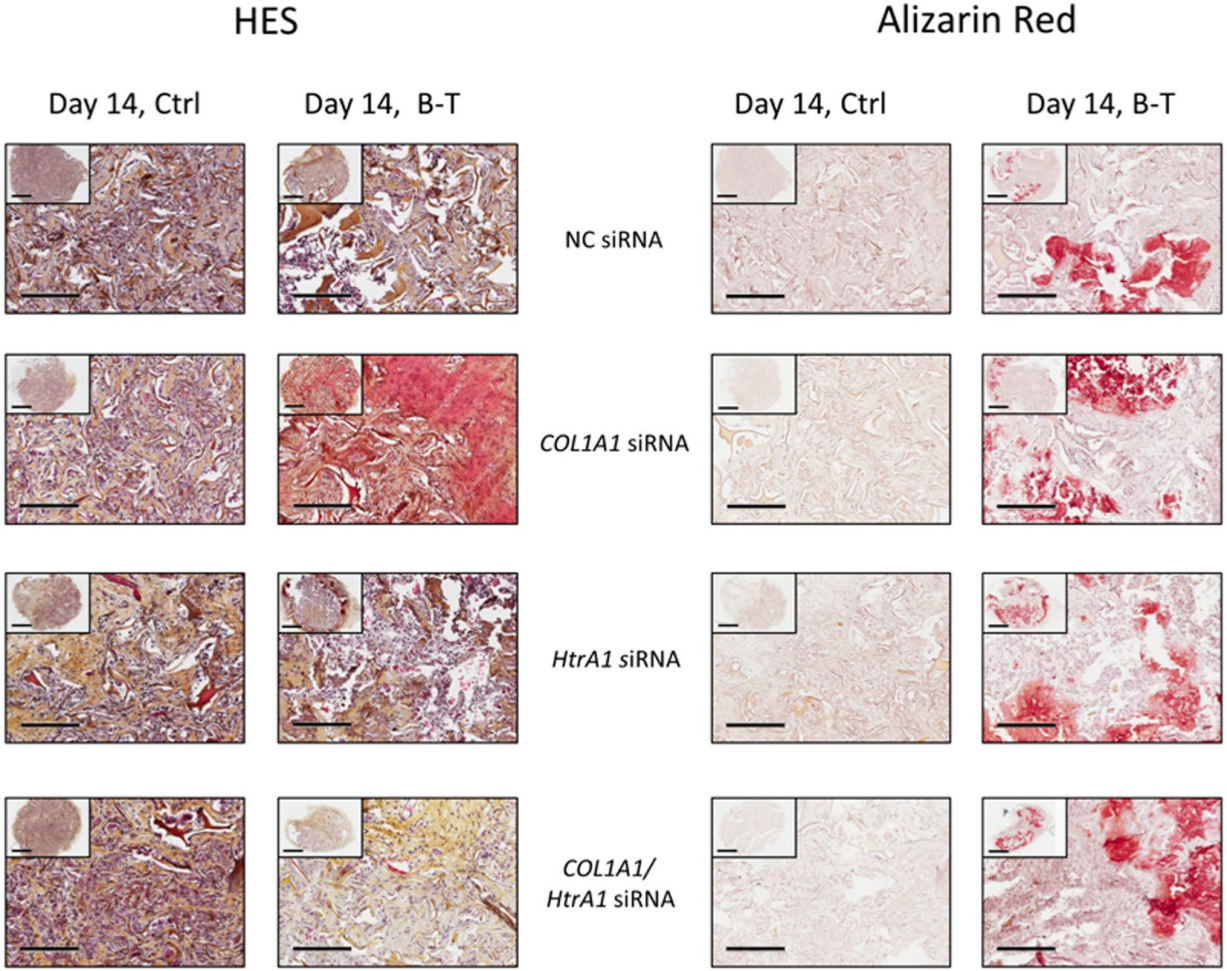
Histological evaluation of ectopic transplants. hBM-MSCs were cultured under hypoxia, with or without BMP-2/TGF-β1 (B-T), and transfected with NC, *COL1A1*, and/or *HtrA1* siRNAs in type I collagen sponges for 14 days, as described in Fig. 6. They were then implanted subcutaneously in *nude* mice and monitored for 28 days, as described in Materials and Methods. HES and alizarin red stainings of the constructs were then performed. Images shown are representative of the experimental results obtained from two of three independent experiments. Scale bars: 1 mm and 200 µm.

The sponge constructs, obtained after 14 days of culture, were implanted subcutaneously in nude mice for 28 days and then analyzed by histology to evaluate the fate of our collagen scaffold *in vivo* (Fig. 8). HES staining showed a large number of cells within a relatively homogeneous collagen network for untreated cells, regardless of the siRNA treatment. The ECM was much more disrupted for BMP-2/TGF-β1-treated cells. This matrix had an osteogenic phenotype for all BMP-2/TGF-β1-treated cells, as shown by alizarin red staining. Such calcification was obtained for two of the three strains of hBM-MSCs used.

In conclusion, our 3D culture model with BMP-2/TGF-β1 treated hBM-MSCs was not effective in producing a cartilage-like matrix in subcutaneous implantations, as the chondrocyte phenotype obtained *in vitro* evolved towards an osteoblast phenotype *in vivo*.

## Discussion

MSCs are considered to be an alternative to chondrocytes in ACI. In this study, we used hBM-MSCs from aged OA patients with the objective to produce a hyaline cartilage-like matrix in a type I/III collagen sponge scaffold *in vitro*. We obtained the best specific chondrocyte phenotype using the combination of BMP-2 and TGF-β1 under hypoxia. We improved the differentiated phenotype with the addition of either *COL1A1* siRNA alone or the combination of both *COL1A1* and *HtrA1* siRNAs, but the sponge constructs failed to form stable ectopic cartilage-like tissue after subcutaneous implantation *in vivo*.

Most studies of BM-MSCs have used cells from healthy donors. Here we used cells from aged OA patients. We first focused on their immunophenotype. These cells had particular features concerning their surface markers. The isolated cultured BM-MSCs did not correspond to a single phenotypic population. They were uniformly negative for markers of the hematopoietic lineage, including CD14, CD34, CD45, and CD64. The expression of HLA-DR (MHC class II) was variable depending on the donor, with the cells derived from only 1/3 of the total population not significantly expressing HLA-DR. hBM-MSCs are not normally antigen presenting cells, and would not be expected to express HLA-DR unless treated by interferon-*γ* ^3, 33^. However, HLA-DR expression was already been described in mesenchymal progenitor/stem cells of human BM^34^ and the use of bFGF during the amplification phase could induce low HLA-DR expression^35^. Donor age, disease, and drug treatments may also influence its expression and explain variability of HLA-DR expression between donors. In contrast, the total population did not significantly express the stem cell marker CD146. Such low CD146 expression has already been described in BM-MSCs from healthy donors^34^ and arthroplasty patients^36^. In addition, CD146 negativity may suggest chondrogenic potential in adult stem cells^37^. The expression of CD90 was globally positive (all >70%), but varied between strains as described for BM-MSCs^38^. The variability of cell surface CD90 levels and low positivity of some strains may be associated with a loss of immunosuppressive activity^39^, as suggested by the HLA-DR positivity for a portion of the strains described above. We observed an increase of CD29 and CD105 expression throughout the passages suggesting an enrichment in the MSC population. BM-MSCs were positive for CD73 and CD44^38^, as expected. They also expressed nucleostemin mRNA, a marker of proliferating stromal stem cells in adult human BM^40^, but not the mRNA of three pluripotent factors, OCT4, NANOG, and SOX2, expressed in embryonic stem cells^41^. We found that the proliferative capacity of aged OA donor-derived MSCs was reduced as previously described^42^, despite the presence of FGF-2 which could enhance their proliferation^43^. Finally, we found that the multilineage potential of hBM-MSCs was maintained until elevated passages (P5), establishing their stem cell nature^4^, despite their low proliferation potential and particular immunophenotype. Indeed, we had an heterogeneous MSC population rather than a pure MSC population in particular for CD29, CD90 and HLA-DR expressions. Unfortunately, few studies have examined the benefits of cell selection criteria for cartilage engineering applications. Whereas CD105 enrichment using a magnetic separator seemed not to influence the multilineage potential of BM-MSCs, the high expression of CD29 seemed necessary for chondrogenic differentiation^44^. Others found that CD29 + /CD90 + selected MSCs from rat bone marrow displayed a significantly reduced capacity for osteogenic/adipogenic differentiation^45^. Further investigations would be required to link phenotypic profiles of the donors with chondrogenic potential, but some authors suggested that maintaining cellular heterogeneity with BM cultures facilitates MSC differentiation^45^.

There is conflicting data concerning the effect of age and disease stage of OA donors on the chondrogenesis of BM-MSCs. The chondrogenic capacity of MSCs obtained from OA patients has been reported to be reduced^42^, whereas others found that BM-MSCs from rheumatoid arthritis and OA patients showed chondrogenic potential similar to that of healthy donor-derived BM-MSCs^46^, and that their chondrogenic differentiation was independent of age and OA etiology^47^. As in most studies, the authors used a traditional pellet culture system to differentiate cells in the presence of TGF-β3. They did not investigate the effects of oxygen, whereas oxygen tension is known to influence their osteogenic, chondrogenic, and endochondral phenotype^1, 18, 19, 48^.

In our study, we cultured hBM-MSCs in a collagen scaffold and compared the effects of oxygen tension on their chondrogenesis in the presence of TGF-β1 and/or BMP-2. We used type I/III collagen sponges that we have previously successfully used for chondrocyte redifferentiation with BMP-2^28, 30^. We showed that this collagen scaffold, not extensively used in the form of a sponge for MSC chondrogenesis, also effectively induced the differentiation of aged OA donor-derived BM-MSCs to the chondrogenic lineage, but only in the presence of a member of the TGF-β superfamily. In particular, we found that hBM-MSCs underwent chondrogenesis only in the presence of TGF-β1 or both BMP-2 and TGF-β1. In addition, BMP-2 amplified the chondrogenic effects of TGF-β1.

Our data are in agreement with the findings of Weiss *et al*., who demonstrated that BMP-2 alone was not sufficiently inductive for chondrogenic differentiation of BM-MSCs obtained from OA patients in a pellet culture model^16^. In contrast, others showed that BMP-2 alone was effective in three-dimensional aggregate or high-density micromass cultures of BM-MSCs obtained from healthy donors^14, 15^. This suggests that the chondrogenic capacity of OA patient-derived MSCs was reduced, as described by Murphy *et al*.^42^, and that they need a more robust induction of chondrogenesis. We also showed that BMP-2 at a concentration of 50 ng/mL had neither hypertrophic, nor significant osteogenic and catabolic effects until 21 days of culture, as already described in other systems^13, 28^.

TGF-β1-induced chondrogenic differentiation of hBM-MSCs was also associated with an increase in *COL10A1*, *MMP-13*, and *HtrA1* mRNA levels. As reported by others, we found that TGF-β1 alone is a potent inducer of BM-MSC chondrogenesis^9, 10^ and hypertrophic maturation of MSCs^6, 14^, but is not an inducer of the final osteoblastic maturation. Additionally, up-regulation of HtrA1 expression by TGF-β1 has already been demonstrated in chondrocytes^49^.

Many publications show that BMP-2 enhances TGF-β1- and TGF-β3-mediated chondrogenic differentiation of various culture systems^12–15, 50^. We showed that BMP-2 amplified the TGF-β1-induced up-regulation of the major chondrocyte specific gene, i.e. *COL2A1* and its spliced forms, regardless of the oxygen tension. The combination of BMP-2 and TGF-β1 also induced the greatest increase in *ACAN* mRNA levels. BMP-2 slightly increased *COL10A1* mRNA levels induced by TGF-β1. However, BMP-2/TGF-β1 treatment only slightly induced the expression of a low molecular-weight form of type X collagen. These data are in agreement with the results of Murphy *et al*. who found that type X collagen was undetectable by immunohistochemistry after BMP-2/TGF-β1 treatment in two-week-old aggregate cultures of hBM-MSCs, whereas TGF-β1 alone, or in combination with BMP-2, induced *COL10A1* gene expression^14^. We found that BMP-2 counteracted the effect of TGF-β1 on *HtrA1* expression and that BMP-2/TGF-β1 treatment decreased the synthesis of HtrA1 protein, strongly induced by culture in sponges. We also observed a down-regulation of HtrA1 synthesis by BMP-2 in BMP-2/TGF-β1-treated hUCB-MSCs (not shown).

Low oxygen tension (5% O_2_) has been found to stimulate the differentiation of stem cells into the chondrogenic lineage and, at the same time, suppress the hypertrophy of stem cells and chondrocytes^18–20^. In contrast, Cicione *et al*., using commercial differentiation media, demonstrated that severe hypoxic conditions (1% O_2_) inhibited adipogenic, octeogenic, and chondrogenic differentiation of hBM-MSCs from three OA donors^22^. Reduced oxygen tension (2% O_2_) also inhibited chondrogenesis and osteogenesis in adipose-derived MSCs cultured in micromass with TGF-β1^21^. Here, we drove chondrogenic differentiation of hBM-MSCs using TGF-β1 or BMP-2/TGF-β1, both under normoxia (21% O_2_) or hypoxia (3% O_2_). We showed that low oxygen tension improved the chondrogenic potential of hBM-MSCs, demonstrated by the induction of *COL2A1* mRNA and its spliced isoforms. Hypoxia also decreased their hypertrophic maturation and HtrA1 expression. We found that hypoxia decreased TGF-β1- and BMP-2/TGF-β1-induced *COL10A1* mRNA levels only about 10 times. Nevertheless, these findings are in agreement with other studies indicating that the improvement of the chondrogenic potential under hypoxia was accompanied by a concomitant suppression of *COL10A1* expression^18, 48^.

We found that the steady-state levels of *COL1A1* mRNA, the major marker of chondrocyte dedifferentiation, were relatively stable for all treatments and oxygen tensions used. Hypoxia appeared to decrease the mature forms of type I collagen protein induced by BMP-2/TGF-β1. It is generally accepted that low oxygen tension inhibits type I collagen expression in redifferentiated chondrocytes^1, 20, 28^, but whether this is true for MSC chondrogenesis is still a subject of debate^19, 48^. Here, we achieved the best chondrogenic potential with the least hypertrophic maturation by BMP-2/TGF-β1 treatment under hypoxia, but the expression of the fibroblastic marker, type I collagen, persisted. Moreover, HtrA1 was still expressed in differentiated BM-MSCs, although BMP-2/TGF-β1 treatment under hypoxia decreased its expression. This is why we used an siRNA to suppress type I collagen expression, and another targeting *HtrA1* to prevent the degradation of BMP, the TGF-β receptors, and ECM proteins. Our mRNA and protein analyses confirmed the efficiency of our siRNA strategy. We observed specific responses of the siRNAs on their own targets, and a combinatory effect of BMP-2/TGF-β1 and *HtrA1* siRNA on the inhibition of HtrA1. The inhibition of HtrA1 by its specific siRNA may result in the persistent effects of BMP-2 on HtrA1 downregulation. We previously used the combination of BMP-2, hypoxia, *COL1A1*, and/or *HtrA1* siRNAs to improve the hyaline matrix nature of the neo-cartilage formed by redifferentiated chondrocytes cultured in type I collagen sponges, both *in vitro* and *in vivo* ^30^. Here, our siRNA strategy with BM-MSCs allowed us to simultaneously reduce the synthesis of the protease HtrA1 and of type I collagen, in favor of the production of type II collagen, with no induction of other proteases. Another group showed that gene therapy, as a strategy to abolish type I collagen expression, could be useful in chondrogenesis^23, 51^. The authors demonstrated that the use of a combinational adenoviral vector encoding TGF-β3 and an shRNA targeting type I collagen promoted the chondrogenic differentiation of synovium-derived MSCs, as well as inhibiting the formation of fibrocartilage. Instead of using a viral vector and shRNA, known to induce stable knockdowns, we used siRNAs to induce transient knockdown, avoiding a gene therapy approach.

Moreover, the improvement in chondrogenic potential with RNA interference was accompanied by a concomitant induction of *COL10A1* and *ALP* mRNA levels (without induction of *MMP-13* and *OSTEOCALCIN* mRNA levels), despite a hypoxic environment. Although type X collagen protein was not clearly expressed, these results reveal an increased risk of hypertrophy and osteoblastic maturation, which was confirmed by the *in vivo* study at an ectopic site. Thus, we observed premature calcification of the BMP-2/TGF-β1-induced cartilaginous matrix as previously described for BM-MSCs from OA donors cultured in pellets with TGF-β3^52^. This calcification was not observable with sponge constructs obtained with BMP-2/TGF-β1-treated UCB-derived MSCs^31^. This unstable phenotype may be due to a predisposition of BM-MSCs to progress towards osteogenesis, the heterogeneous nature of the MSC population, or the age of the donors^17^. Several studies suggest that hypertrophic maturation obtained with BM-MSCs from OA donors can be down-regulated using parathyroid hormone related protein (PTHrP) *in vitro*, but one showed that resistance of the pellets to *in vivo* calcification was not achieved at an ectopic site^16, 53^. Other laboratories suggest that extended chondrogenic induction *in vitro* (>12 weeks) is necessary for hBM-MSCs to retain stable cartilage characteristics in a non-chondrogenic environment *in vivo* ^54^, whereas we performed chondrogenesis for only 2 weeks *in vitro* to obtain clinically relevant results. There are therefore few possible ways for controlling the hypertrophic maturation of MSCs from elderly OA donors: the implantation of constructs in an articular site, which is not conceivable in mice, and inhibiting the upstream hypertrophy with a Runx2 siRNA.

In conclusion, we successfully induced chondrogenic differentiation of a heterogeneous population of hBM-MSCs from aged OA donors, with low proliferative potential, in a collagen scaffold. We obtained the best chondrocyte phenotype using the combination of BMP-2 and TGF-β1 in a hypoxic environment. The chondrogenic lineage was improved using an RNA interference strategy to prevent the synthesis of the major marker of fibrocartilage and a protease overexpressed during the OA process. Nevertheless, further studies are needed to control the hypertrophic maturation of BM-MSCs *in vivo* and to verify their immunosuppressive potential. Our study suggests that BM from OA patients would not be the best source of MSCs for cartilage tissue repair strategies, but it provides new insights for improving the differentiated chondrocyte phenotype to develop new methods of ACI therapy in humans.

## Electronic supplementary material

Supplementary information
