# Supplementary material for: Enhanced chondrogenesis of bone marrow-derived stem cells by using a combinatory cell therapy strategy with BMP-2/TGF-β1, hypoxia, and COL1A1/HtrA1 siRNAs
Source: Sci Rep. 2017 Jun 13;7:3406. doi: 10.1038/s41598-017-03579-y (PMC5469741; doi:10.1038/s41598-017-03579-y)
Supplement: Supplementary file 1 — Supplementary information [file 41598_2017_3579_MOESM1_ESM.pdf]

Enhanced chondrogenesis of bone marrow derived-stem cells by using a combinatory cell therapy strategy with BMP-2/TGF-β1, hypoxia and *COL1A1/HtrA1* siRNAs

Florence Legendre, PhD[1], David Ollitrault, PhD[1], Tangni Gomez-Leduc, PhD[1], Mouloud Bouyoucef, PhD[1], Magalie Hervieu, PhD[1], Nicolas Gruchy, MD-PhD[1,2], Frédéric Mallein-Gerin, PhD[3] , Sylvain Leclercq, MD-PhD[1,4], Magali Demoor, PhD[1], and Philippe Galéra, PhD[1*].

[1] Caen Normandy University, France; UNICAEN EA4652 MILPAT (Laboratoire Microenvironnement Cellulaire et Pathologies), MIPDF Team (Microenvironnement des Pathologies Dégénératives et Fibrotiques); UFR de Médecine, Université de Caen, Caen Cedex 5, CS14032 Caen, France.
[2] Laboratoire de Cytogénétique Prénatale, Service de Génétique, CHU Caen, France.
[3] Institute for Biology and Chemistry of Proteins, CNRS, UMR 5305 Laboratory of Tissue Biology and Therapeutic Engineering, Université Claude Bernard-Lyon 1 and University of Lyon, France.
[4] Service de Chirurgie Orthopédique, Clinique Saint-Martin, Caen, France.
[*] Corresponding author: philippe.galera@unicaen.fr

ADDITIONAL MATERIALS AND METHODS

Scanning electron microscopy (SEM)

hbM-MSCs cultured in collagen sponges were fixed for 24 h with 2.5% glutaraldehyde in PBS at 4°C, and washed in 0.1 M phosphate buffer, pH 7.4. Samples were post-fixed with 1% osmic acid for 2 h, washed and dehydrated in increasing concentrations of ethanol. They were then critical point-dried and coated with a platinum layer for SEM observations (JEOL 6400F, France) at CMABio (SFR ICORE 4206, University of Caen Normandy, Caen, France).

A
B
C
D

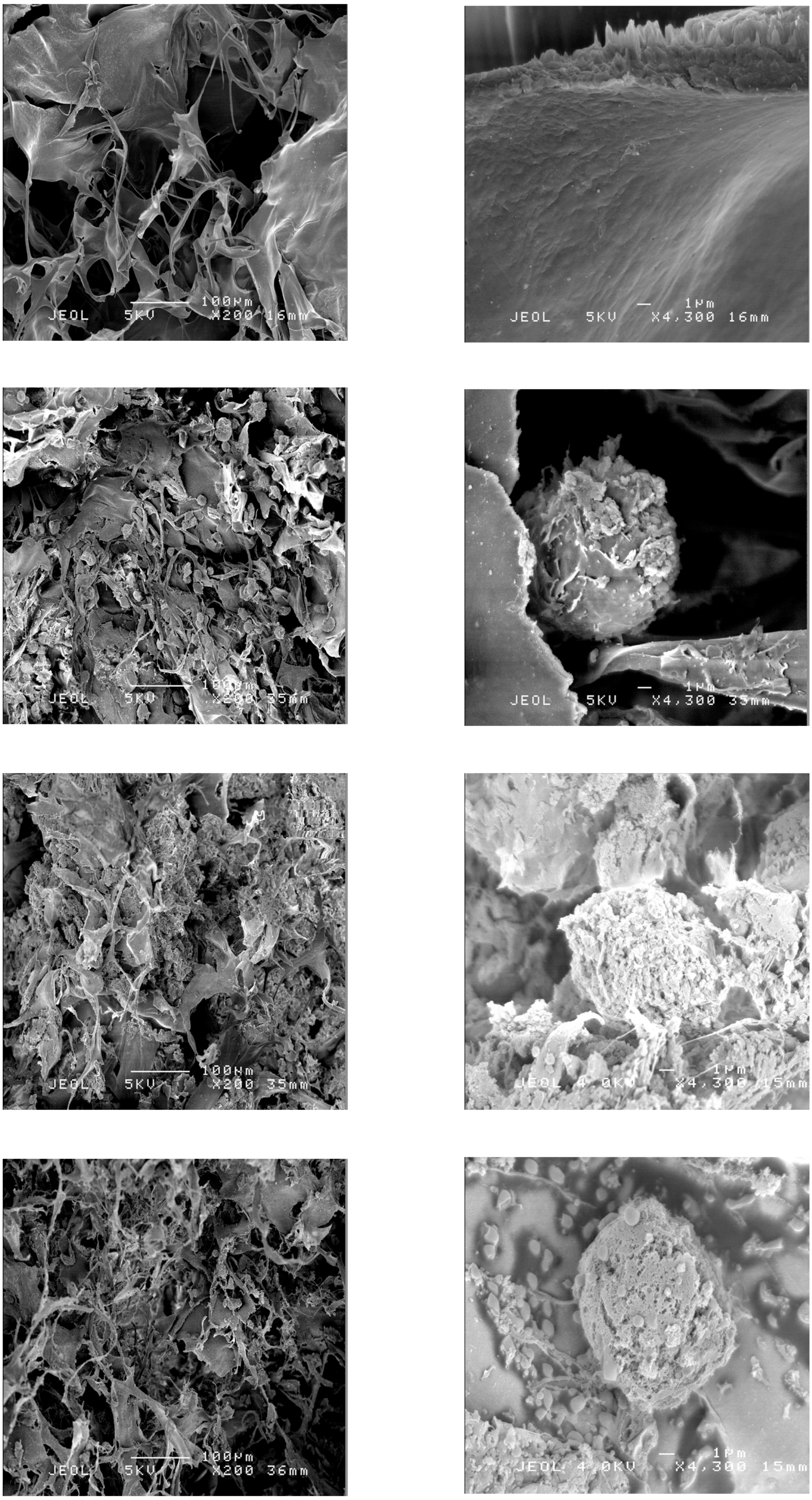
100µm
JEOL 5KV X200 16mm
1µm
JEOL 5KV X4,300 16mm
100µm
JEOL 5KV X200 35mm
1µm
JEOL 5KV X4,300 35mm
100µm
JEOL 5KV X200 35mm
1µm
JEOL 4.0KV X4,300 15mm
100µm
JEOL 5KV X200 36mm
1µm
JEOL 4.0KV X4,300 15mm

Fig. S1. Scanning electron micrograph of hBM-MSCs cultured in a type I collagen sponge. hBM-MSCs were cultured in a type I collagen sponge for 1 h (day 0) or for 7 days under normoxia or hypoxia (n=1). SEM observations in the internal area of the sponge were then performed. Left panels, original magnification x 200 and scale bar: 100 µm. Right panels, original magnification x 4300 and scale bar: 1 µm. **A.** Micrographs of a type I collagen sponge without cells. **B.** Micrographs of a sponge seeded with MSCs taken at day 0. **C.** Micrographs of a sponge seeded with MSCs cultured under normoxia taken at day 7. **D.** Micrographs of a sponge seeded with MSCs cultured under hypoxia taken at day 7.

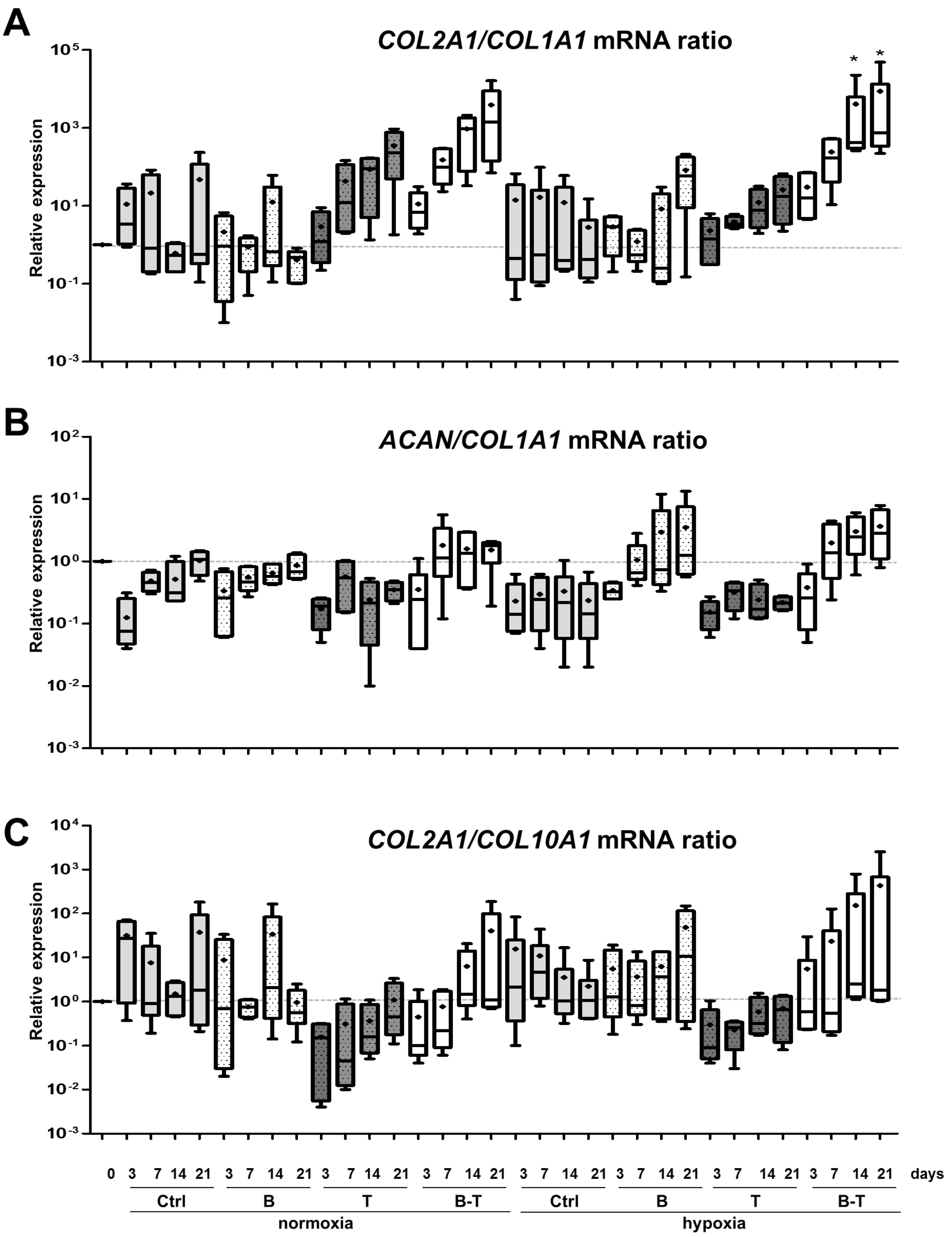

Fig. S2. Effect of culture conditions on chondrocyte differentiation indexes. hBM-MSCs were cultured in type I collagen sponges for 3, 7, 14, and 21 days, as described in Fig. 3. The levels of mRNA encoding type I collagen (*COL1A1* mRNA), type II collagen (*COL2A1* mRNA), type X collagen (*COL10A1* mRNA), and aggrecan (*ACAN* mRNA) were measured

using real time RT-PCR with specific primers. All the results were normalized to *RPL13a* mRNA and the expression of each gene relative to that of undifferentiated hBM-MSCs at day 0 was determined. We then determined the *COL2A1/COL1A1*, *ACAN/COL1A1*, and *COL2/COL10A1* ratios presented as box plots representing the results of independent experiments in triplicate (n=4). Statistically significant differences between the undifferentiated cells at day 0 and treated cells are presented and were determined using the Kruskall-Wallis test (*P < 0.05).

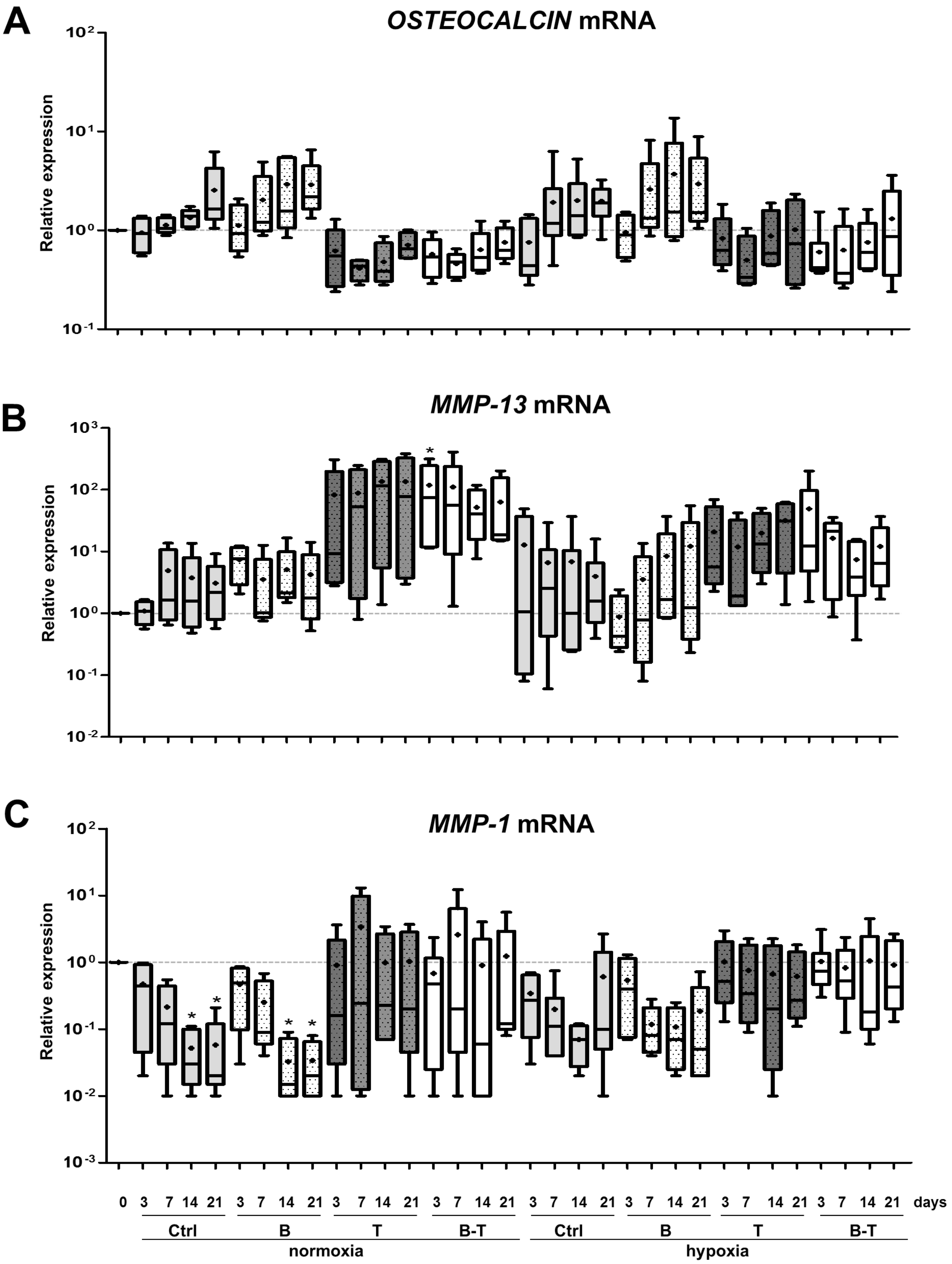

Fig. S3. Effect of oxygen tension, BMP-2 and/orTGF-β1 on the mRNA levels of catabolic markers and an osteogenic marker. hBM-MSCs were cultured in type I collagen sponges for 3, 7, 14, and 21 days as described in Fig. 3. The levels of mRNA encoding MMP-1 (*MMP-1* mRNA), MMP-13 (*MMP-13* mRNA), and osteocalcin (*OSTc* mRNA) were

evaluated using real time RT-PCR with specific primers. All the results were normalized to *RPL13a* mRNA and are presented as the expression of each gene relative to that of undifferentiated hBM-MSCs at day 0. Box plots represent independent experiments performed in triplicate (n=4). Statistically significant differences between the undifferentiated cells at day 0 and treated cells are presented and were determined using the Kruskall-Wallis test (*P < 0.05).

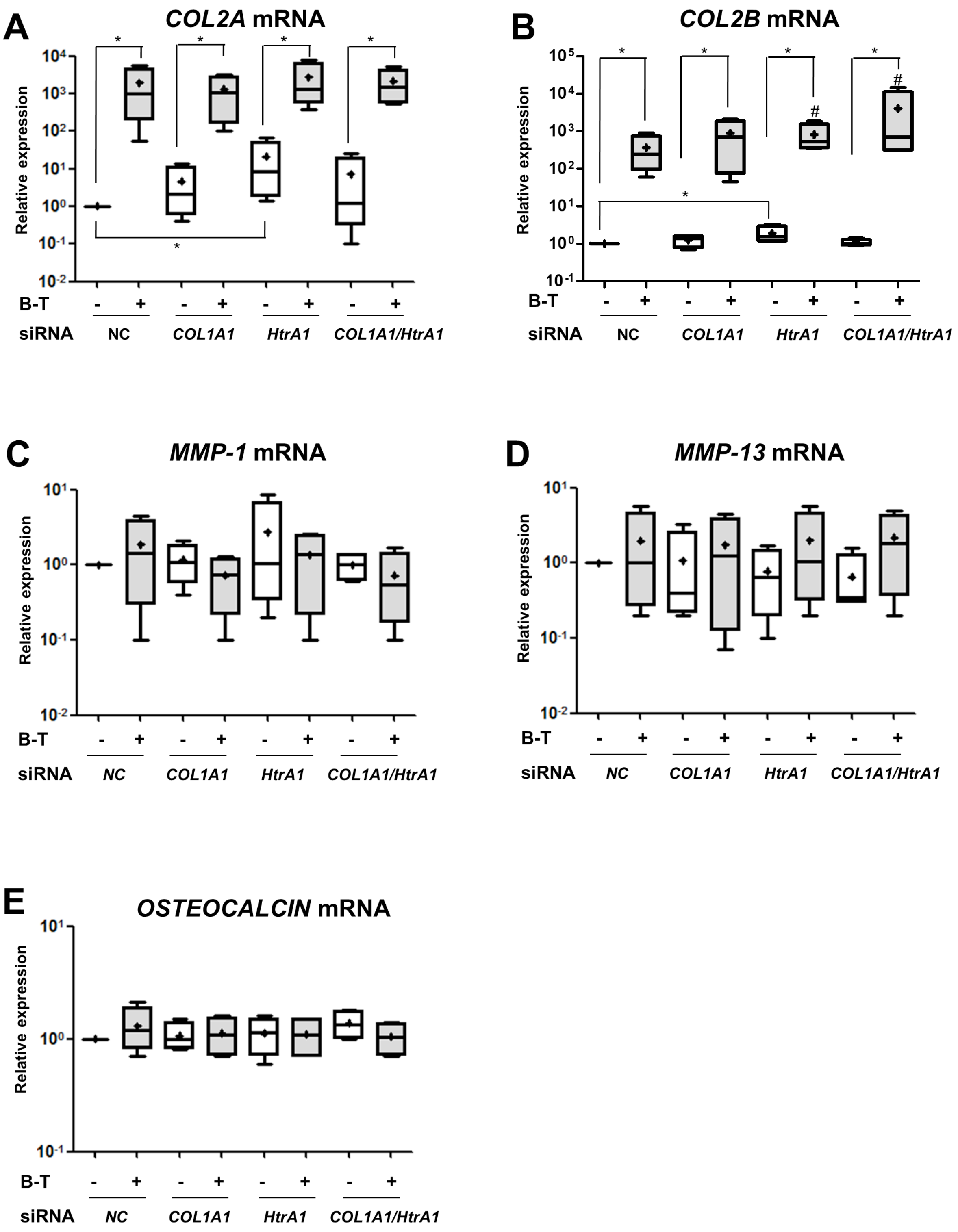

Fig. S4. Effect of *COL1A1* and/or *HtrA1* siRNAs on the mRNA level of splice variants of type II collagen, catabolic markers, and an osteogenic marker. hBM-MSCs were cultured in type I collagen sponges for 14 days under hypoxia, with or without BMP-2/TGF-β1 (B-T), and transfected with NC, *COL1A1* and/or *HtrA1* siRNAs, as described in Fig. 6. Relative

mRNA expressions of type IIA collagen (**A**), type IIB collagen (**B),** MMP-1 **(C)**, MMP-13 (**D**), and osteocalcin **(E)** were obtained as described in Figs. 3 and S3. All results were normalized to *RPL13a* mRNA and are presented as the expression of each gene relative to that of untreated NC cells. Box plots represent independent experiments performed in triplicate (n=4). Statistically significant differences between the untreated NC cells and the various treatments were determined using the Kruskall-Wallis test (#$P < 0.05$). Statistically significant differences between untreated cells and BMP-2/TGF-β1-treated cells, NC siRNA-untreated cells, and other siRNA-untreated cells, and between NC siRNA BMP-2/TGF-β1-treated cells and other siRNA BMP-2/TGF-β1-treated cells were determined using the Mann-Whitney U test (*$p < 0.05$).

Table S1

Primers used in this study.

Sequence name	Sequence (5'→ 3') (F: Forward; R:Reverse)
RPL13a primers	**F:** GAG GTA TGC TGC CCC ACA AA<br>**R:** GTG GGA TGC CGT CAA ACA C
COL2A1 primers (total form)	**F:** GGC AAT AGC AGG TTC ACG TAC A<br>**R:** CGA TAA CAG TCT TGC CCC ACT T
COL2A primers	**F:** TGC AGG ATG GGC AGA GGT ATA<br>**R:** GAG GCA GTC TTT CAC GTC TTC AC
COL2B primers	**F:** CCG CGG TGA GCC ATG A<br>**R:** TTT GGG TCC TAC AAT ATC CTT GAT G
COL2B taqman probe	**FAM**-CCA GGA TGT CCG GCA ACC AGG A-**TAMRA**
COL1A1 primers	**F:** CAC CAA TCA CCT GCG TAC AG AA<br>**R:** CAG ATC ACG TCA TCG CAC AAC
COL10A1 primers	**F:** AAA CCA GGA GAG AGA GGA CCA TAT G<br>**R:** CAG CCG GTC CAG GGA TTC
Aggrecan core protein primers	**F:** TCG AGG ACA GCG AGG CC<br>**R:** TCG AGG GTG TAG CGT GTA GAG A
Osteocalcin primers	**F:** CGG TGC AGA GTC CAG CAA A<br>**R:** GGT AGC GCC TGG GTC TCT TC
ALP primers	**F:** AGC CCA GAG ATG CAA TCG<br>**R:** CTA TCC TGG CTC CGT GTC C
MMP-1 primers	**F:** GAA GCT GCT TAC GAA TTT GCC G<br>**R :** CCA AAG GAG CTG TAG ATG TCC T
MMP-13 primers	**F:** AAG GAG CAT GGC GAC TTC T<br>**R:** TGG CCC AGG AGG AAA AGC
HtrA1 primers	**F:** GGGACTGGTCGTGTTTGTGC<br>**R:** CATTGACCTTTGGGTGCTGACT
OCT4 primers	**F:** ACC CAC ACT GCA GCA GAT CA<br>**R: CCA CAT CGC CCA GCA GCT TGG**
SOX2 primers	**F:** GTC ATT TGC TGT GGG TGA TG<br>**R:** AGA AAA ACG AGG GAA ATG GG
NANOG primers	**F:** CCC CAG CCT TTA CTC TTC CTA<br>**R:** CCA GGT TGA ATT GTT CCA CCA GGT C
GNL3 primers	**F:** ATG ACC TGC CAT AAG CGG TAT<br>**R:** CTT AAA GGG AGC ACT GTT TGG A

Fig.S5 GAPDH-hyoxia-exposure1

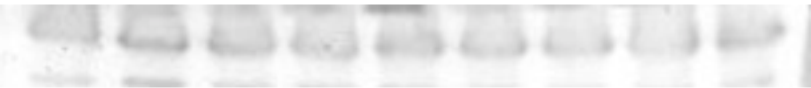

Fig.S5 GAPDH-normoxia-exposure1

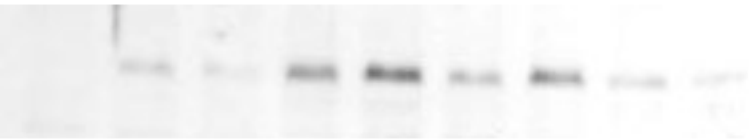

Fig.S5 HtrA1- hypoxia-exposure 1

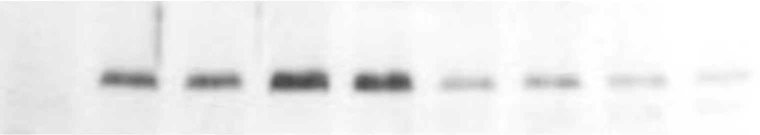

Fig.S5 HtrA1- normoxia-exposure 1

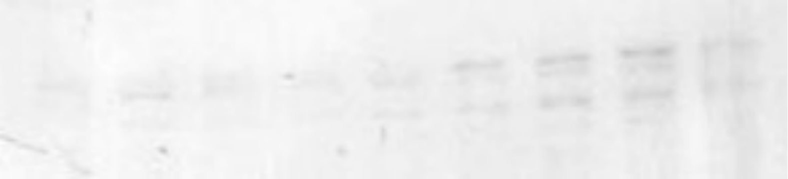

Fig.S5 Type I collagen- normoxia-exposure 1

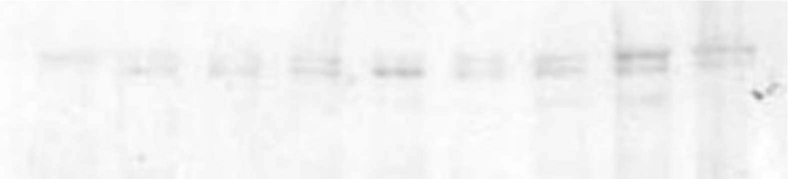

Fig.S5 Type I collagen-hypoxia-exposure 1

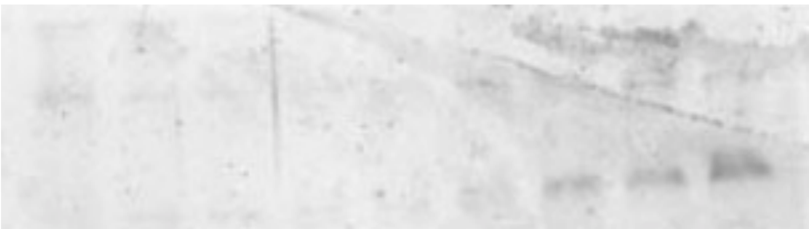

Fig.S5 Type II collagen- normoxia-exposure 1

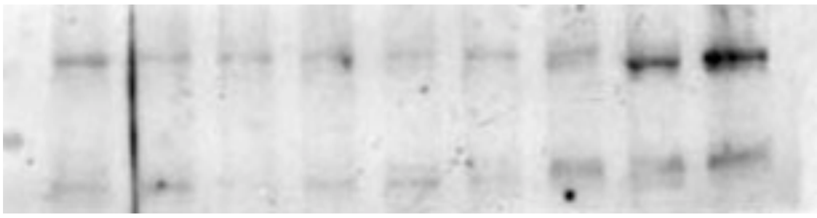

Fig.S5 Type II collagen- normoxia-exposure 2

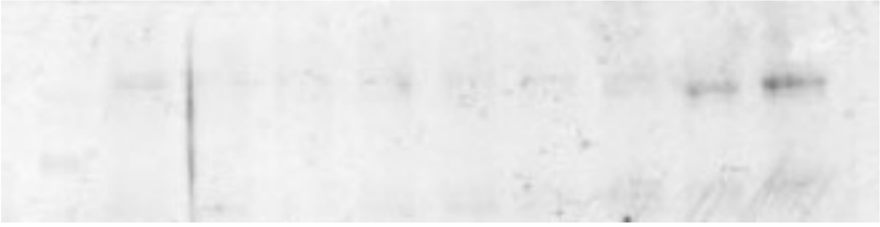

Fig.S5 Type II collagen-hypoxia-exposure 1

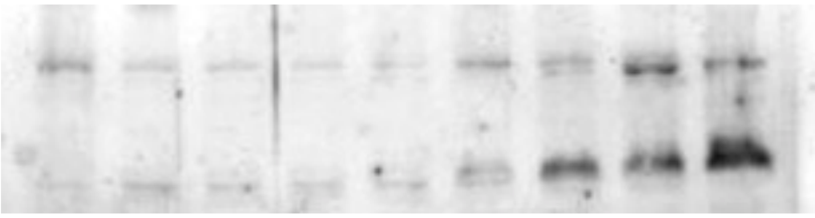

Fig.S5 Type II collagen-hypoxia-exposure 2

Fig. S5. Effect of culture conditions on collagen and HtrA1 synthesis. hBM-MSCs were cultured in type I collagen sponges for 3, 7, 14, and 21 days, as described in Fig. 5. Protein extracts were analyzed by Western-blotting for type I, II, and X collagen, and HtrA1 *versus* GAPDH. Different exposures of the films are provided.

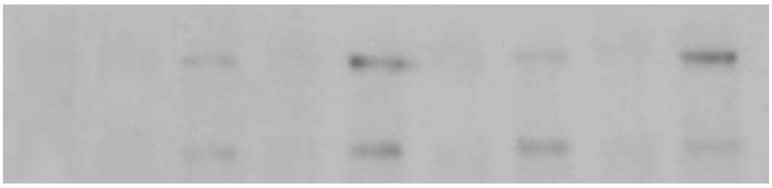

Fig.S6A Type II collagen-exposure1

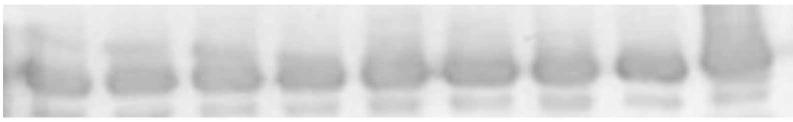

Fig.S6A GAPDH-exposure1

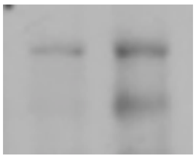

Fig.S6B Type II collagen-exposure1

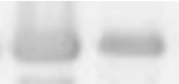

Fig.S6B GAPDH-exposure1

Fig. S6. Effect of *COL1A1* and/or *HtrA1* siRNA on collagen and HtrA1 synthesis. hBM-MSCs were cultured under hypoxia with or without BMP-2/TGF-β1 (B-T), and transfected with NC, or *COL1A1* and/or *HtrA1* siRNAs in type I collagen sponges for 14 days, as described in Fig. 7. Protein extracts from treated-MSCs, undifferentiated MSCs at day 0 (D0), and a macroscopically healthy zone of human OA cartilage were analyzed by Western-blotting for type I, II, and X collagens, and HtrA1 *versus* GAPDH. Different exposures of the films are provided.
